# Mask sociology as a way of action theory: Voices of the face mask among social individuals in the COVID-19 masquerade in South Korea

**DOI:** 10.1371/journal.pone.0293758

**Published:** 2023-11-02

**Authors:** Jae-Mahn Shim

**Affiliations:** Department of Sociology, Korea University, Seoul, Korea; University of Massachusetts Lowell, UNITED STATES

## Abstract

South Koreans are susceptible to the medical face mask against the COVID-19 pandemic. Meanwhile, their mask practices are intriguingly laden with contradictions and inconsistencies. This study accounts for this puzzle by expanding two sociological frontiers: the sociology of action (i.e., action theory of agency and individuality) and the sociology of the mask. Drawing on action theory, it stresses that contradictions and inconsistencies reveal the nature of individuals as social individuals and develops a typology of social individuals during the current pandemic (i.e., atomists, collectivists, and dualists). For mask sociology, it amplifies that any mask practices are conceptualized as a masquerade involving multiple elements for individuality and proposes a theory of mask multivocality that appreciates the ways in which masquerade the social drama becomes concretized. With this two-pronged conceptual innovation, it first demonstrates a patterned relationship between social individuals and mask multivocality. Dualists take more voices from the mask than atomists or collectivists. Dualists take the most contradictory voices as well. Second, it shows that Koreans who take more meanings from the mask reveal not only more vulnerability but more transformative power amid the current pandemic. Demonstrating the promise of mask sociology for the action theory of individuality, it ultimately argues that individuals as social performers often reveal themselves as mask-wearers who become as transformative as they are vulnerable. While this model is founded upon the recent pandemic, it ramifies in political and cultural events that various face coverings accompany.

## Introduction

South Korea is one of the East Asian countries known for the public’s early and ready acceptance of the surgical face mask against the COVID-19 viruses, compared to the arguable resistance in Western countries [1–3]. A closer day-to-day investigation, however, points to nuanced tensions, confrontations, and dramatic transitions underneath the apparent acceptance.

It was not until the central government announced the severity of the virus in February, 2020 that people started having interests in the face mask and market prices for a piece soared 5–7 times from half a US dollar to around three dollars [4]. Before then, there was a surplus of masks in drugstores from limited suppliers. Putting a mask on the face in the streets had not been a popular idea during the latest infection event of MERS (Middle East Respiratory Syndrome) in 2015 [5]. MERS had contracted 186 people and had killed 38 nationwide over half a year, recording the second highest death rate among the OECD countries [6]. According to a national survey, only 15% of the population had ever worn the face mask during the MERS outbreak [5]. Some Koreans had argued for the necessity of wearing the mask for protection [7], while many others had argued against the mask that, in their eyes, had spread unnecessary fear for the disease and jeopardized economic and social activities [8–10]. People had often projected quizzical looks onto mask-wearers in the streets, delivering an unfounded belief that these mask-wearers were either weak or overreacting to the virus. It was different in 2020. Upon the governmental confirmation of domestic cases of the disease (“Wuhan influenza” as called at the time) and alleged protection effects of the mask, masks started being sold out everywhere. The mask enjoyed overnight popularity, and the central government had to rely temporarily on the state-controlled mask rationing. It was an unmistakable and yet arbitrary transition from neglect in 2015 to near-obsession in 2020, unaccountable solely by the incremental behavioral adaptation to seasonal yellow dusts and particulate pollutants in the air [3, 11].

Furthermore, during the public’s normative support for the mask against COVID-19, breaches are frequent, and different social groups have different standards on when and where to wear the mask in practice [12]. Police departments across the country report violent confrontations between an individual insisting on mask-wearing and another resisting it, although the government has already set a nationwide rule in May [13]. Mask-wearers are always concerned with learning local rules not to wear it in personal or business meetings. Non-wearers are concerned with when they should submit to wearing it in different contexts. As political legitimacy is at stake, central and municipal governments have had to discreetly decide when and how to design legal mandates for mask-wearing. Private shops and businesses are no exception to this delicate game of legitimacy-claiming for their chances for business. Individuals, too, want to be savvy enough to don and doff the mask properly one moment after another to be taken as responsible citizens and free individuals at once. While supporting mandatory mask-wearing, many Koreans turn infuriated by the government’s additional legal enforcement in mid-November 2020 that collects a fine of 100,000 won (about 90 US dollars) for violating the mandate.

These contradictions and tensions constitute the empirical surprise of this study. What does the dramatic transition from neglect to obsession (or the coexistence of neglect and obsession within only a few years) reveal about Koreans? What is the nature of Koreans’ widespread acceptance of the mask which is fragile in practice? What is this oxymoron? What does Koreans’ ready submission to government mandates for mask-wearing along with their paradoxically casual breaches of these mandates refer to? All the while, Koreans are much occupied and concerned with the mask. Some may argue that the surprise over these contradictions is endemic only to the Korean (or East Asian) context and not generalizable to other countries. However, it is worth noting that Westerners are similarly intriguing, as they fervidly verbalize rejection against face-covering and yet wear the mask in practice [14–16].

This empirical surprise resonates with a particular sociological tradition. Ambivalence and contradictory coexistence in human practice are the central concerns of the sociology of action and agency, or action theory [17–20]. Human agency is usually defined in the literature as the capacity for an individual to engage the social world [21]. It spans the Weberian rationality of multiple kinds for a subject’s mastery over life matters in the world [22–24]; the Parsonian human efforts to orient personal needs to institutional requirements [25]; the Swidlerian pragmatic problem-solving with multiple cultures in action [26, see also the Peircean pragmatism in 27]; the Foucauldian subjectivity-making among multiple power relations [28, 29]; the Maussian existential security/totality among diverse ultimate forces in the world [30]; the Bourdieusian ontological complicity (and enmity) with the world [31]; the Merleau-Ponty’s embodied harmony (and disharmony) with the world [32: 142].

So far as existential human experiences are concerned, agency is represented in these theories to originate in supra-individual as well as individual elements, such as alters as well as ego; social institutions as well as individual motivations; non-human animals and artifacts as well as humans; deities as well as humanities. Along with this expanding list of supra-individual elements, individual elements of agency become multiplicated and imploding, including corporeality and embodiment as well as mentality; emotions and affects as well as reason. Hence come multiplicity, contradiction, and ambivalence into the notion of agency. Agency as such needs to be approached in such a way that it is comprehensive, pragmatic, and existential enough to be true of real human experiences. In this context, a group of contemporary studies finds action and practice to be a proper sociological subject for agency [17–19, 26, 33, 34]. Thus, action theory, or an action-focused theory of human agency makes the contemporary theory of human agency proper.

Within this action theoretical appreciation of multiplicity and contradictions, the empirical surprise about the COVID-19 face mask in Korea can be duly answered. In brief, multiplicity and contradictory coexistence within agency in action point to the fact that individuals are “social individuals” [35]. That is to say, the capacity of an individual resides in supra-individual and even transcendental [25] relations and entities, in addition to individual elements such as different members of a person (e.g., body, mind, and soul). Both individual and supra-individual elements are relevant for the existential experiences of a person, while these elements contradict as well as coalesce with one another. Out of this multiplicity of agency in action, therefore, the matter of agency for a person becomes the matter of how that person relates one element of existence with another, no matter what the process is called in the literature. In the process, the person may reveal contradictions and mismatches as well as harmonies and congruences, which are the different manifestations of the person being an agentic social individual. From this perspective, this study posits that inconsistencies and contradictions in the mask practices among Koreans reveal that Koreans engage in practicing agency as social individuals.

The remaining task is, therefore, to elaborate on social individuals in the specific practices of the mask. To this purpose, this study combines another insight from action theory. The extent to which people combine supra-individual and individual elements to construct their agency in action varies from one person to another. Drawing on the literature of varieties of social individuals, this study proposes a typology of social individuals that differentiates three types: atomist social individuals who acknowledge only individual elements for agency; collectivist social individuals who stress only supra-individual elements; dualist social individuals who stress both. It then specifies these different types of social individuals among Koreans by leveraging a pair of the COVID-19-specific questionnaire items. This specification contributes to action theories’ conception of the sociality of individuality (i.e., social individuals) [35] by elaborating the conception into different types amid empirical reactions to COVID-19.

Among these reactions to COVID-19, the face mask is situated to manifest richer details about people being social individuals than other instances. As a material object, the face mask demarcates individual elements of agency (e.g., a distinct body and mind) and supra-individual elements (e.g., the corona virus and other people) from one another. At the same time, it enables juxtaposition and mutual orientations among these elements. The mask signifies an individual body that is distinct from and yet interacts with the corona virus and other human bodies in greater or smaller tensions. The mask’s very location at the intersections of individual and supra-individual elements with this double entendre (e.g., separation and connection at once) provides mask-worn people with the foundation for different and often contradictory ways in which they signify the mask in practice.

To reveal these diverse and contradictory mask practices systematically, therefore, this study develops a theory about voices of the mask that lays out the semiotic contours of double entendre, drawing on the sociology and anthropology of the mask and face-covering [30, 36–38]. This theory of mask multivocality conceptualizes the mask as referring to a dynamic process of multiple elements (i.e., a masquerade, the social drama) that contradict and coalesce with one another for agency and individuality. According to this conceptual frame, this study demonstrates that the face mask against COVID-19 signifies multiple and contradictory meanings, such as both alters and ego; physical protection, psychological relief, and symbolic social ritual at once; both life adversity and life betterment during the pandemic. It then demonstrates a patterned relationship between the types of social individuals and mask multivocality. The dynamic and inconsistent practices of the mask among Koreans are patterned into those of atomist, collectivist, and dualist social individuals. What the current COVID-19 masquerade reveals, it argues, includes three different ways in which social individuals make and remake their individuality in Korea during the pandemic.

Based on the patterned association between mask practices and social individuals, this study agrees with existing theories of action that agency and individuality consist in multivocality. Subsequently, it addresses a further question on what multivocality means in more specific terms by investigating concrete experiences accompanying the face mask. It shows that Koreans ascribing more voices to the mask are more susceptible to new promises as well as damages from the pandemic. It thus argues not only that multivocality is accompanied by vulnerability and transformative power, but that vulnerability is closely knit with transformative power. While the former statement addresses what the multivocality of humanity consists of, the latter suggests that it is the vulnerability in humanity that enables the transformative power in human agency. In reverse, vulnerability is implied in the transformative and creative power of humanity. Humans are as transformative as they are vulnerable. These statements mark the ultimate contribution that the Korean experiences of the COVID-19 masquerade make to action theory.

In all, this study proposes a general theory of social individuals in masquerade. It provides a set of answers to the initial empirical surprise about Koreans’ ambivalent and contradictory practices about the mask. The ambivalence and contradiction are revealed in the varying multivocality of the mask, which reflects the condition of individuals being social individuals of different kinds. When social individuals become dualists than atomists or collectivists, they take greater multivocality from the mask. The greater multivocality individuals take from the mask, the more likely they reveal both vulnerability to the world’s damages and transformative power amid these damages. These answers have several theoretical implications. Firstly, they renew action theory. This study argues that the creativity of human action, or the riches of action as creative power that individuals hold [17, 39], is not only based upon the multiple dimensions of action that individuals navigate but on such multi-dimensional action’s ready vulnerability to shocks and damages. Human creativity and its transformative power lie in vulnerability. Secondly, these answers promise a new avenue for pursuing action theory (i.e., an action theory of the mask). Whether it be the medical face mask, political protesters’ face-covering, or eye masks at cultural events, the mask is one of the social locations that action theory should look at for the dynamics of action and agency. Thirdly, this action theoretical approach to the mask renews the sociology of the mask. The existing mask literature implies multiple meanings embedded in the mask and face-covering, without clearly qualifying the extent to which these meanings are activated in context. By providing a model that specifies how the multivocality of the mask is differently manifested, this study complicates the existing literature. Fourthly, the answers have implications for the external literature on Korean or Asian individuality. To these effects, this study analyzes responses from a nationally representative sample of Koreans during the early months of the COVID-19 pandemic in 2020. In the following, theoretical affordance precedes empirical findings.

## Action theory and social individuals

### Ambivalence and contradictory coexistence in action

Complexities and inconsistencies of human action have been the central subject of recent developments in action theory [17, 18, 33]. Sociological theories of action can be first understood as attempting to provide the micro-level voluntaristic foundation of individual action that simultaneously serves macro-level social systems and institutions [20]. Often dubbed a structural functionalist account, this attempt envisions wise individuals who perceive the requirements of supra-individual systems and institutions as well as their personal needs and motivations, against early and still recurring pathetic accounts that depict indigenous and sub-cultural groups of people as ignorant or unscientific individuals not knowing own motivations and supra-individual consequences of these motivations. The only trouble with this legitimate Parsonian counter-argument lies in its exaggerating wise individuals to the point of being readily wise and omniscient individuals who always oversee the system of multiple sub-systems, let alone various personal needs and motivations [40]. This exaggeration is not quite the same as the moderate conception of only would-be-wise and intelligent individuals. Considering his early mentioning of individual “efforts” to properly control and coordinate means to ends and one value-end with another value-end [25], however, this trouble does not need to be overstated any further [21: 965–6]. Instead, heed needs to be paid to the structural functionalist’s fundamental acknowledgment of plural social institutions and plural (and often conflicting) individual motivations in action that necessitate efforts for control, for example, among medical professions addressing the matter of death as well as life [41] and myths as well as science [40]. It attributes the potential complexities and contradictory coexistence in action to the fact that meaningful individual action does not consist only in a person’s egoist motivations but supra-individual institutions and norms in the world.

Secondly, sociological theories of action originate in the discipline’s venerable interest in agency [18, 42: 1–21]. Agency can be defined as the capacity for individuals to engage the social world [21], causal forces/mechanism [43, 44], causes [19], and ultimate forces and existences in the world [30]. These definitions attest to a close and yet less emphasized link between the sociological subject of agency and the philosophical matter of ultimate existences in the universe [17, 45]. Notably, the pragmatist approach in philosophy that attempts to address these existences with the focus on tangible human experiences overlaps squarely with the sociological interest in human agency on an empirical dimension. This overlap highlights that individual human agency comprises expanding instances of existence, such as a person’s corporeal body and immaterial mind; emotions and cognitive reasoning; alters and ego; nonhumans and human actors; deities and humanity. Agency is located potentially among these multiple existences together.

Recent treatments in general sociological theories comprehend this complexity and contradictory coexistence in agency with renewed interests in action as a whole [18, 19, 33]. While these theories each stress creativity [17], reflexive intersectionality [33], temporality [21, 46], social aesthetics [19], and aesthetical interests [47] as the nature of the complexity and contradiction in action, earlier studies have addressed the same matter under other sociological subjects, such as the rationalization and elective affinities among various values and individuals [see 22, 23 for a comprehensive review of Max Weber’s take on affinities, 48], the dual process of governmentalization and subjectification of different powers [29, 49], total social services or the individual–social totality revealed as a gift [39], techniques of the body and the body of techniques [50], a toolkit of settled and unsettled cultural tools [26], the habitus organization of multiple habiti in practice [34], liminality both between and betwixt life experiences [51], the assemblage of humans and non-human actors [52], bricolage [53], embeddedness [54, 55], and ambivalence [56, 57].

In all, multiplicity, contradictory coexistence, and ambivalence in agency and action are inevitable to the extent to which individual experiences of existence are founded not only upon different members of a person (e.g., mind, body, and soul); they are also based on various supra-individual entities, involving other individuals (and their different members), social institutions (e.g., schools and workplaces), roles, non-human actors (e.g., artifacts, bacteria, and viruses), and supernatural powers (e.g., spirits and divinities). The ambivalence and contradictory coexistence in agency reflect the very fact that individuality exists within and without the individual mind and body, as schematically represented in a binary formula that personhood encompasses “the acting self (toward an object)” and “the object self” [58: 421] and in another conception that mental life (i.e., freedom) is composed of both inter-personal individuality and intra-individual personality [59].

### Types of social individuals

To properly consider these individual and supra-individual aspects that individuals are exposed to, it is apt to devise a flexible notion of “social individuals” that draws on the Meadian understanding of primary sociality residing in all individuality [17, 35]. What is fundamental to individuals who exist as social individuals is that there are inevitable and vital tensions in becoming social individuals in practice. These tensions are epitomized by ego–alter agonism (i.e., agonistic coexistence) versus antagonism (i.e., antagonistic negation) [39], and ego’s domination versus elimination of alters for ego’s existence and recognition in Hegel’s master/slave dialectic [60]. Depending on how these tensions play out, social individuals can become differentiated from one another. Studies have indeed revealed potentially different types of individuals, depending on the extent to which agency and individuality are co-constructed with and co-present among individual and supra-individual elements.

This paper proposes three types of social individuals to consider varying developments of these tensions: atomists, collectivists, and dualists. In conceiving of this typology and proposing “dualist” social individuals as one of the types, the following elaboration is especially relevant [61: 541]. “We are constantly seeking ultimate forces, fundamental aspirations, some one of which controls our entire conduct. But in no case do we find any single force attaining a perfectly independent expression, and we are thus obliged to separate a majority of the factors and determine the relative extent to which each shall have representation. (…) An action that results from less than a majority of fundamental forces would appear barren and empty. (…) Human life cannot hope to develop a wealth of inexhaustible possibilities until we come to recognize in every moment and content of existence a pair of forces, each one of which, in striving to go beyond the initial point, has resolved the infinity of the other by mutual impingement into mere tension and desire.” Thus, Simmel proclaims, “man has ever had a *dualistic* nature.”

#### Atomists

Although there are various ways in which social individuals construct their agency and individuality at the intersections of individual and supra-individual elements, atomists place their agency only in the individual elements and not in the supra-individual ones. In such a way, atomists tend to develop a single-dimensional understanding of agency-generating motives and rationality and find it challenging to develop and organize various kinds of motives and rationality simultaneously.

Whether it is called the capacity for individuals to engage the social world [21] or the Weberian rationality for individuals to wield the mastery over life matters [22], agency and individuality among atomists are placed outside the will of supra-individual powers, such as God’s Providence [33: 64], and instead placed within individual elements, such as motives and preferences that are pre-known to each individual. To the extent to which individuality is placed among pre-known and fixed motives and preferences, it is outside of dynamic supra-individual relations with other human actors [62] and non-human natural actors such as animals [63], artefacts [64, 65], and viruses [66], not to mention supernatural powers [67]. Atomists do not usually consider supra-individual entities since they are irrelevant to their pursuit of individuality. If they ever consider these entities, which seem to them to derive only from other individuals, they deem these entities to be hazardous to or competitive with their individuality. Furthermore, atomists’ individuality defined by pre-known motives is placed even outside of new motives emerging opportunistically in the course of their own action and practice in the world. If any new motives emerge, they are not given independent recognition but quickly reduced to pre-known motives ([e.g., the "individualist emergentist" view in 68]). In another word, agency and individuality among atomists precede sociality and externality of any kinds.

To the extent that preferences are outside of supra-individual elements that are divine, spiritual, material, and social-relational, preferences that one atomist holds are strange to those that another atomist has. Except for rare occasions where these two different sets of preferences happen to be cognizant of each other a priori, it is difficult for either atomist to see bridges between one’s own preferences and those of the other. An atomist rarely relativizes one’s pre-given preferences while being dynamically informed by others who hold preferences that are strange to oneself. Therefore, atomists tend to develop certain types of rationality that seem only irrational to others (i.e., the Weberian iron cage of rationalization), and these types tend to remain fixed [i.e., "the fixation on different concepts of rationality" in 18: 271]. More problematically, therefore, atomists are unlikely to resolve existential paradoxes of rational ego confronting irrational alters; ego being both rational (e.g., scientifically rational) and irrational (e.g., sensually rational) simultaneously; both rational and emotional; both gentle and brutal [e.g., the famous contrast between Adam Smith and Thomas Hobbes regarding the state of nature in 69]. If they ever hope to avoid these paradoxes, atomists must know in advance the ways in which paradoxes do not emerge as paradoxes from the very beginning. Furthermore, requirements of social institutions and collectivities are likewise strange and untouchable to atomists, unless these requirements are somehow already known to and internalized by them. Whenever social institutions and collectivities emerge that are not pre-known to atomists, atomists run into insuperable trouble coordinating these institutions with their own motives.

Atomists frequently emerge in the literature under different other names. Their focus in practicing individuality upon isolated individual elements is recognized in such notions as “undersocialized” individuals who are innately either gentle (as in Smith’s *The Wealth of Nation*) or brutal (as in Hobbes’ *Leviathan*) in an invariable manner [69]; a workman who knows in advance what is in one’s cultural toolkit and uses these cultural tools ingeniously at times of “settled lives” [26]; “boss”-like political leaders who are charged with personal motivations for power domination with little interest in public goods and legitimacy [70]; political challengers who hold interests distinct from those of incumbents and attempt to uproot the whole dominant system for their non-communicable interests [71]; biologists living with “certainty” and no doubt despite unsettling laboratory findings that go against the canonical Mendelian laws of biology [27]. Atomists’ single-dimensional fixation to one kind of rationality as the only foundation of individuality is recognized in the notion of individuals who see varieties of rationality as “nothing but” one fundamental rationality (be it material or cultural) [72]. Their trouble in organizing varieties of rationality is highlighted in the conception of individuals who are caught in destructive conflicts between “hostile worlds” each of which is run by a distinct logic of rationality, and thus avoid any contamination across these worlds [72] and instead seek purity of each world set apart from one another unpolluted [73].

#### Collectivists

Collectivists place their agency and individuality in the supra-individual entities and not the individual ones. So long as they do not seek their individuality at the intersections between individual and supra-individual entities, they share with atomists a single-dimensional and static approach to agency-generating institutions only in a slightly different manner. Therefore, it becomes impossible for collectivists to truly appreciate diverse foundations of institutions and subsequently organize them for their agency. They only hope these diverse institutions to be already organized under an overarching logic and be effective foundations for their individuality and agency.

Unlike atomists who look inside themselves and identify sources of individuality from within, collectivists find few internal sources and instead attend to the world outside of ego. Ego is full of complexities and uncertainties to collectivists, including unclear motives and unstable preferences. In the face of complex and unclear motives, these individuals fulfill the task of reducing these uncertainties for their existential security by imitating what fellow individuals do who seem to be cognate with them and by listening to what others require them to do. Taken to a higher level, collectivists locate their individuality among what organizations and social institutions ask them to function as. To the extent to which these institution- and system-level functions and roles reduce the complexity and uncertainty that a lone individual feels in front of the world [18: 272–273], these functions and roles become effective sources of agency and individuality.

This shift from the ego-centered to the alter-centered perspective or from the individual viewpoint to the collective one is what defines collectivists. Relatively unstable individual motives and preferences that are not sanctioned by demands and requirements from outside have no place in collectivists’ individuality. Therefore, when a collectivist’s individuality defined by the demand of one social institution meets a contradictory demand of another social institution, the collectivist’s individuality fails unless the two social institutions are already organized by an orchestrating demand of a third institution or a meta-system [25]. Unclear and evolving individual motives have nothing to do with this process of orchestrating. Collectivists with effective individuality are readily conscious of a meta-system, or an over-arching system that comprehends all other (sub-)systems. To this extent, it is futile and unnecessary for collectivists to perceive and know all the differences and complexities of various institutions, that is to say, various supra-individual aspects of individuality; they already (assume to) know, thus foreknow, the ultimate requirement of a meta-system and base their individuality on this static and ready omniscience and foreknowledge. Individuality becomes fixed and univocal in terms of the requirement of the one and only meta-system. Otherwise, individuality among collectivists is one of the contradictory and fractured nature caught between different needs of institutions that are not organized by any meta-system.

Collectivists with similar characteristics are called by different names in the literature: “oversocialized” individuals who are readily knowledgeable of and adamantly loyal to collective rules [69]; a workman who “is used” by (not “uses”) cultural tools such as ideologies and big ideas at moments of “unsettled lives” [26]; “judge”-like political leaders who are concerned only with public goods and legitimacy and not personal ambitions to be a boss [70]; political incumbents who maintain the current dominant system for their distinct interests against challengers [71]; biologists living with all “certainty” and no doubt about the established Mendelian laws among fellow biologists, without witnessing any laboratory experiments that cast doubts to the laws [27]; individuals who live out various logics of supra-individual “worlds” in a reductionist or confrontational manner [72]; purity-seekers who live only within the system set apart from non-system and disorder [73].

#### Dualists

Unlike the preceding two types of social individuals, dualists place their agency and individuality in both individual and supra-individual elements. They draw individuality from pre-known personal motives as well as unknown motives dynamically evolving in action and inter-action; scientific rationality as well as sensual rationality; cognition as well as emotions; personal motives as well as inter-personal and institutional/systemic requirements; sub-systemic institutional logics as well as meta-systemic necessities. As such, dualists tend to develop multi-dimensional understandings and practices of individuality that do not only span across individual and supra-individual elements but across various kinds of individual elements (e.g., rational and irrational motives; varieties of rationality) and across multiple supra-individual entities (e.g., human and non-human alters; positivist-material and transcendental-real alters and institutions). To this extent, individuality and agency are constantly evolving and expanding from one shape to another, depending on the opportune context of action and practice. Unlike atomists and collectivists, individuality among dualists is hardly fixed, pre-known, or pre-required unless action and practice are put to a halt; it is flexible, unknown, and instantly realized and emerging. In this sense, individuality among dualists is renewable for long; it is (re-)generative, (re-)creative, and (re-)constitutive of itself thanks to its multiplicity.

When individuality is renewing itself among dualists, it has far-reaching implications for the development of supra-individual institutions. Individuality of a person that is at the beginning inclusive of some of supra-individual institutions and systems is in turn generative of other novel institutions and systems when it renews itself within the person in practice. That is because dualists’ individuality is always connected to the supra-individual level. In this sense, dualists’ practice of individuality is liberating and emancipating to a greater extent than that of atomists or collectivists. The individuality of atomists is fixed and finite only to the individual level, while that of collectivists is confined to the supra-individual level and thus not easily amendable by individuals.

At the same time, dualists’ individuality is fixed and finite to the extent that it involves many sub-systems and the existence of a meta-system, whether material or meta-physical, that overrides all these sub-systems. This finiteness is what gives dualists an anchoring and regulating sense in the practice of individuality, in addition to a renewing sense of generative and creative powers in individuality. The only curiosity and uneasiness that dualists hold, which is one of the very sources of generative powers of their individuality, is ultimately targeted toward realizing the substantive content of an evolving meta-system.

Dualists are called by different names in the sociological literature, such as “embedded” individuals who are both brutal and gentle, both solitary and relational, and both economic and social [69, 74]; a workman who does not only use but is simultaneously used by cultural tools [26, 75]; a Sphinx-like “multivocal” political leader who is both a self-interested boss and a public-minded judge at once [70]; “socially skillful” political incumbents who pursue their interests in the current system only to the extent to which they can persuade challengers that the system is congruent also with challengers’ interests, and “socially skillful” challengers who cannot overturn the dominant system immediately and yet sustain themselves within the system and their hope for a different future [71]; biologists living with both “doubt and certainty” at the encounters between novel individual findings in experiments and a collective knowledge system of Mendelian biology [27]; “relational/connectionist” [72, 76] or “ritualizing” [77] individuals who relate personal intimacy with formal money transactions in tensions; individuals who seek both within-system purity and cross-system pollution at once [73].

#### Hypotheses

Based on the typology of social individuals, this paper develops testable empirical hypotheses to specify their existence during the current pandemic in S. Korea. It addresses how many are atomists, collectivists, and dualists in a society at a certain point in time. Given the distinct traits of three types of social individuals, furthermore, several patterns will hold true. The following set of hypotheses purport to illustrate the validity of the typology of social individuals by showing that it is aligned well with some existing measures of individualism, collectivism, and the sense of control in life.

HYPOTHESIS 1–1.—For example, collectivism, defined as an attitude that values collectivities more than individuals [78], is expected to increase the chances of becoming collectivists and dualists and lower the chances of becoming atomists.

HYPOTHESIS 1–2.—On the contrary, individualism, the opposite attitude, defined as an attitude that gives precedence to individuals over collectivities [78], is expected to increase the chances of becoming atomists and dualists and lower the chances of becoming collectivists.

HYPOTHESIS 1–3.—Lastly, given that there are a variety of individual and supra-individual entities in which people locate their agency and control powers over life (i.e., the locus of control) [79, 80], people who locate their agency at more loci are more likely to be dualists than atomists or collectivists who place agency on a relatively limited number of entities (either individual or supra-individual entities but not both).

## Voices of “the mask”

People put the mask on the face, such as surgical face masks, eye masks, veils, and head-coverings, as a matter of securing agency and individuality at the intersections between ego and the surrounding world of human and nonhuman alters. In doing so, social individuals who exist in different combinations of individual and supra-individual entities practice the mask in different manners. This paper elaborates on this relationship between types of social individuals and ways of mask-wearing to the effect of illustrating that mask-wearing is a matter of social drama (i.e., masquerade) [30, 36–38, 81] so much as individuality is a matter of social performance discussed in the previous section of social individuals.

In a Goffmanesque perspective, a mask means one of many faces that individuals manage to put on for the presentation and making of the self [36]. Goffman draws on a view that denies that “substance exists for the sake of appearance (or vice versa), and faces for the sake of masks (or vice versa);” the view instead stresses that “all these phases and products are involved equally in the round of existence” [81: 132]. In this perspective, masks are relevant to the self so much as faces are to it. While the self is presented and acted out with multiple selves, such as frontstage and backstage selves [36, 82], face-work (i.e., another Goffmanesque notion of the self-making) [37], through which individuals perform multiple faces and yet manage to “have (and not lose) a face,” involves not only multiple faces but even masks. In this sense, face-work appears to be indistinct from any mask-work.

Indeed, predating Goffman’s highlight on these links among face, mask, and self, Mauss earlier conceptualizes the problem of the self as “masquerade” [30]. According to the Maussian perspective, the mask in itself is the self and individuality which can be properly formulated as a whole “course” [30: 22] or a social process of self-making [83: 87, 84] that involves individual and supra-individual entities (i.e., masquerade the social drama). That is why the mask is often found to be so dysfunctional, unnatural, and exaggerated (i.e., ‘symbolic’ vs. instrumental in the Parsonian sense [25]) at the same time as it functionally fits the face of wearers and materially reflects the surrounding nature (e.g., feathers of birds and tongues of fish) [38].

First, people do not wear the mask when alone; they wear it when with other human and non-human alters. People do not always wear it when with alters but only when the surrounding alters pose unusual threats or chances for existential security. People wear it when they feel like reconstituting their existence in the eyes of others. In this way, the mask points to individuals being social individuals. Second, when the mask is deemed relevant for individuality, the ways in which people wear the mask vary a lot. Some oppose it unconditionally for securing individuality, while others accept it unconditionally for the same purpose; still others bother to strike a balance between opposition and acceptance, and remain committed to both, while it is more or less challenging to do so in different contexts. Third, this process of securing individuality amid sociality via the mask reveals varying, sometimes paradoxical, aspects of the mask. As a material object, the mask intervenes between ego and the surrounding world by setting up a dividing barrier (either for ego or the world); other times, it sets up a transmitting conduit (either from ego to the world or from the world to ego). As a symbolic object, the mask intervenes for individuality by signifying the presence and absence of ego (i.e., effecting the ego that is absent covered by the mask and yet bodily present underneath it); it simultaneously signifies the presence and absence of the surrounding supra-individual world (i.e., effecting the world that is revealed and regarded by the mask and still often dismissed beyond it). The following elaborates each of these.

### A wall and a conduit for ego

The mask on the face is consequential for the practice of individuality. The act of face-covering concerns the fact that people found individuality upon various members of the corporeal body including the face and extra-facial body parts (e.g., critical organs and functioning limbs) [32, 50]. In addition, facial body parts (e.g., eyes and mouth) have a direct relationship with the “discovery and development” [85: 254] of non-bodily emotion and cognition which are as consequential as the physicality of the face [37]. At the same time, to mask facial body parts or not has bio-physical implications, if controversial, for extra-facial bodily subsistence (e.g., the functioning of respiratory organs).

Therefore, it is no wonder that people assign varying meanings to the mask. People believe that mask-wearing infringes on individuality since the mask intervenes as a wall that covers up and hides the facial bodily existence of individuals away from the surrounding world. In addition, this dividing wall is deemed to preclude emotion and cognition that are usually discovered and developed in relation to the face. This worry stands legitimate even though, by covering up ego’s face, emotion, and cognition, the same mask may produce the possibility for others to discover and develop their bodily and non-bodily existence. To the extent to which social individuals like atomists do not regard others’ existence as relevant to their own individuality, they take the mask as threats to individuality.

By separating ego from the world, all the while, the mask contingently protects non-facial body parts and secures the otherwise vulnerable corporeal existence. People take the mask as an opportunity for individuality to the extent that it prevents corporeal existence from being contaminated and encroached on by external threats, such as the scorching sun onto the fragile human bodies in the desert [86], lustful gazes of men onto Muslim female bodies [80, 87: 75, 88: 305] and the punitive state surveillance over the vulnerable bodies of protesters [89: 267]; in doing so, it ultimately preserves the non-facial bodily aspect of individuality.

Meanwhile, people like atomists do not care about whether their mask-wearing produces any negative impacts upon others’ bodily and non-bodily existence to the extent to which they see others irrelevant to their individuality. If ever, others’ existence in the supra-individual world is often regarded only as the transposition of ego’s individuality through the conduit of the mask. In this sense, people take the mask as an opportunity through which ego’s personal entities are asserted as individuality and further passed as essential elements onto sociality.

The mask works as a wall dividing ego from the world or as a conduit transmitting ego toward the world. In addition, the mask becomes a threat or an opportunity for individuality among atomists. They recognize that the mask can negatively affect one aspect of individuality (e.g., emotion on the face) while it affects another positively (e.g., bodily health). A loss of individuality in one aspect does not necessarily mean the loss of individuality in all the other aspects. People calculate relative gains and losses. If gains exceed losses, they value the mask. If losses exceed gains, they devalue it. Depending on this one-time calculation, the mask attains a particular meaning for individuality. Characteristic in this calculation is the irreversibility and non-renewability of the lost aspects of individuality despite the gained individuality in other aspects. Atomists do not acknowledge that the loss of individuality in one aspect due to the mask (e.g., the negation of face-based emotional existence) that is accompanied by the individuality secured in another aspect thanks to the same mask (e.g., non-facial bodily existence in an enabled, laboring body) can be compensated by a new round of creation (e.g., emotional existence via the very laboring body, if not the face).

First, it is not genuinely appreciated among atomists that the face-based emotional expressions of individuality, such as happiness and pain, are possible thanks to the physicality of the face rather than the face per se. Emotions emerge as valid existence to the extent that they are accompanied by specific physicality, that is, certain muscle movements on the face that people come to call smiling or frowning when no other instances of physicality are afforded, including a slow- or fast-moving laboring body. As far as physicality is concerned, fast- or slow-laboring bodies can alternatively reveal emotions as much as smiling or frowning faces do. However, emotional expressions and developments that are opportunistically related to the face are mistaken among atomists as present exclusively in relation to the face or by themselves without any physical underpinnings. In this limited appreciation, once emotions are blocked by the mask, they are deemed to return only when the face returns free of the mask or when emotions return hopelessly by themselves. In this reasoning, relevant aspects of individuality are delimited to a few that are compartmentalized from one another, rather than those evolving into many that intersect with one another. As a result, atomists do not acknowledge the dynamic process of renewing individuality in which individuality of a certain kind (e.g., a bodily one) can produce individuality of another kind (e.g., a non-bodily one) in unforeseen, emergent ways. They do not acknowledge that non-bodily, emotional existence is not constituted only by facial, bodily existence but other emergent possibilities of bodily existence that are extra-facial, manual, pedestrian, and more. These possibilities are unknown to those atomists who assume that any aspect of individuality is pre-given and essential rather than emergent [68], generative, and developmental in the process [90].

Second, this static and self-restricting view of individuality regarding the mask becomes reinforced among atomists since atomists do not consider the relevance of supra-individual entities for individuality. More importantly, they disregard the mediation of these supra-individual entities that help regenerate individuality on a growing number of aspects. For example, they do not consider the mediation of supra-individual entities through which bodily individuality leads to the making of non-bodily individuality. Non-facial corporeal bodies, or many people who are physically present in one place, first produce sociality of a bodily kind, such as a group of bodies in a metropolis like the one that Simmel observes [59]. Out of this bodily sociality, or the mere existence of many bodies with a ‘blasé’ look on the face, non-bodily individuality (i.e., mentally unique individuals despite all-the-same blasé-faced bodies) emerges unexpectedly by comparison and distinction among a population of corporeal bodies through wears and fashions on these bodies [61]. Atomists overlook that bodily individuality leads to the rejuvenation of lost non-bodily individuality via the mediation of bodily sociality.

In empirical studies, this unique conception of irreversibility and non-renewability of individuality results in aligning the mask, whether it be taken as a threat or an opportunity for individuality, with a relatively limited set of meanings and voices that are symbolic or material. First, studies report that the mask threatens individuality among atomists by covering up and denying ego’s presence. American Muslim women who uphold individual rights in the American South find the hijab, or the headscarf in the Muslim tradition, to oppress femininity (i.e., female individuality) by denying female bodies from the male-dominated social world [87: 83, 91, 92]. This view of the head-covering is sometimes reinforced in the aftermath of the 9/11 attack in 2001, whence the hijab is taken either the implosive suppressor of femininity or the malign, explosive self-assertion of terrorists killing others [93]. Masked individuals, such as terrorists and perpetrators, are granted no legitimate individuality in the mass media [94, 95: 20–21] that reflects the idea of “the utopia of transparency” in which any disguise of identity undermines political legitimacy [89: 266, 96: 97]. Likewise, anonymity refers to deception and exclusive politics rather than honesty and inclusive politics [97]. In a more general context, the mask is taken as a symbolic marker of deficiencies in the individuality proper. Studies of recent experiences against COVID-19 report that the mask is taken as a marker for virus-carrying, weak-and-sick individuals [98].

Second, if the mask is not found to threaten individuality, it is taken by atomists to enhance individuality by separating and protecting ego from the world. The hijab is taken as a means to keep femininity from being breached by the surrounding social world (e.g., lustful gazes of men) imposing on female bodies, and the hijab is for feminine individuality to fully develop apart from sociality [80, 87: 75, 88: 305]. Other times, head-covering and face-covering are in deference to the sun and sand in deserts for the sake of individual survival [86]. Similarly, a historical account [99] on the Japanese’ general acceptance of the surgical face mask against the Swine flu around 2009 finds that the mask is given the meaning of a barrier that separates and protects ego from the dangerous, virus-polluted outer world, carrying out the rising norm of self-responsibility and control for one’s own health rather than fulfilling the often alleged collective obligation to protect the broader community of Japanese society. A similar observation is made on the Japanese’ willingness to wear the surgical mask against COVID-19 [100]. The study conjectures that this willingness is not reflective of the collectivist thinking or group norms [101, 102] but the Japanese’ desire for ‘anonymity-’driven individuality ‘shutting out the crowds around them’ and ‘being shielded from the surroundings.’ This description inclines toward a more symbolic conception that the mask is taken as ‘a form of veil’ shielding the Japanese from ‘the gaze of others’ rather than as a health protection measure. In another Asian context, the first-generation modern Chinese doctors educated the airborne transmission theory of plagues are found to have worn the fabric face-coverings during the 1910–11 plague outbreaks in Manchuria [103] in their sincere hope that the coverings would separate and contain individuals from the surrounding world and guarantee existence. Outside of Asia, a study stresses the generality of this view of the mask by highlighting that plague doctors appearing in Hobbes’ Leviathan wore the beaked mask as the allegedly Leviathan-provided protection wall, material and symbolic, against unknown supra-individual sources of plagues [104].

Third, the mask means a conduit between ego and the world among atomist social individuals. It works as a material conduit through which ego participates and asserts oneself physically in the world or as a symbolic accouterment that is an immediate part of ego. As for the material side, the mask can be a benign tool that enables ego to participate in the otherwise dangerous and unfavorable world. Some Muslim women take the hijab as the great “equalizer” that allows women to join in the otherwise unwelcoming male-dominated social world [87, 88, 105]. Ego’s participation in the world can be sometimes destructive, as in the imageries of terrorist attacks to destroy the external world [93]. For the symbolic side, the mask is taken as a handy bodily prothesis that is designed and stylized by ego to be ego’s extension in the very pre-designed and stylized manner, such as design face masks [100] and designer hijabs [106].

### A wall and a conduit for the world

How the mask becomes consequential for individuality lies not only in its impacts upon bodily and non-bodily aspects of ego but also in its influences on aspects of the supra-individual world surrounding ego. The latter is more the case among collectivist social individuals who take various supra-individual elements of sociality as the sole foundation of individuality. Therefore, collectivists don the mask in such a way that they yield to the prominent duty and necessity to save sociality, apart from which there exists no distinct foundation of individuality for them. As sociality reveals itself in various material instances (e.g., a population of corporeal bodies) and immaterial instances (e.g., a shared sense of collectivity and public authority), people have varying responses to the mask that affects sociality.

The mask separates and protects a body of people who happen to be relatively less unstable and less dangerous from one or more individuals who are more susceptible to the uncertainties and risks inherent to life experiences [107, 108]. For example, in the face of increasing dangers and uncertainties of spreading infectious diseases, a person voluntarily puts on the mask to separate and protect others from the dangerous ego. In doing so, the ego attempts to secure the bodily existence of an uncompromised population of alters which subsequently function as sanguinary material provisions at most and, at least, no deleterious harm for ego’s later attempts to restore its bodily existence. In these maximalist and minimalist senses, the subsistence of others and sociality is the only way in which fluctuating and dangerous individuals solidify and sustain their selves and agency.

The sociality that collectivists turn to for individuality is more than physical. It is often cognitive and affective, such as a shared sense of collective powers and a sense of belonging to supra-individual associations. The mask is one of the conduits through which these powers and associations are revealed and sensed by individuals. For example, by wearing the mask that imitates animal totems or ancestral spirits (i.e., myth-certified constituents of a whole world to be imagined) from the past and is believed to sustain in the future, individual members of a tribal community acquire their unique personal values in the present time [30]. Little attention is paid to who carries the mask or whether the person carries it effortfully or not. In the contemporary scene of COVID-19, the government-certified protective face mask (vis-à-vis improvised home-made face coverings) holds unique symbolic effects mainly because it is qualified by governmental authority and a functioning collective system of science. By wearing the collectivity-certified mask, people ascertain collective powers and, simultaneously, their individual existence. It is unmistakable that people wear the face mask stamped with scientific, organizational, or national-political appellations and symbols, while others wear the ritual mask inscribed with animal totems and temple stamps. In political struggles, protesters wear the mask that has common features sharable among them and enjoy its “uniform”-like effect that provides its wearers with a shared foundation for power (i.e., a collective identity) and a sense of community among the otherwise disconnected individuals [89: 267, 96: 108, 109: 85–86, 110]. In these instances, little attention is given to individuals’ varying difficulties and efforts in wearing the mask [30: 9].

Due partly to this inattention to individual efforts, at the same time, the mask produces adverse effects on the effort to save sociality. While the mask keeps the face-less, masked system of sociality (i.e., an everlasting symbolic system of totemic masks and a population of face-covered corporeal bodies) and reveals the existence of systemic powers, it simultaneously spawns the risk of dissipating the sense of community among mask-carrying individuals by divesting them of personal faces and face-mediated affective expressions of personal efforts tuned to the systemic powers. Masked corporeal bodies which exist faceless in the system of masks tend to be taken as individuals who exist without much emotional or normative commitments toward one another. To the extent that these masked bodies are a peril to sociality of these non-bodily and non-systemic inter-relational kinds, the mask becomes a peril to individuality in the collectivist sense.

Depending on which aspects of sociality are concerned, therefore, the mask becomes an opportunity for, a threat to sociality, or both. However, just like atomists hardly take the mask as both an opportunity and a threat, but only as either the former or the latter exclusively, collectivists do not take the mask’s ambivalence and end up with the same semiotic impoverishment as atomists. This impoverishment results from two related sources.

First, although sociality is multi-dimensional in that it involves corporeal, affective, material, and symbolic aspects, these multiple aspects are not always significant for collectivists. If sociality exists in any aspect that subsequently guarantees individuality, other aspects in which sociality (and thus individuality) further exists are relatively unimportant among collectivists. When the known aspect of sociality is in peril, however, other aspects become significant. Once these multiple aspects are recognized as important, it becomes urgent for collectivists to explain where these still existing aspects of sociality come from while other aspects disappear. Subsequently, they come to see that sociality of one kind (e.g., a population of corporeal bodies) is constitutive of and constituted by sociality of another kind (e.g., a shared sense of community). For example, a population of corporeal bodies usually comes with a shared sense of community. However, the mutual embeddedness at this stage can be static and, thus, fragile. The corporeal kind of sociality at the stage is constitutive of and constituted by the affective kind of sociality on the codified formula that corporeal bodies are naturally accompanied with a shared sense of community. The black box of how the two ever come to accompany each other is left unexamined. Whenever this natural accompaniment is doubted in cases where one sociality sustains while the other disappears, collectivists are clueless about how to put back together these diverging developments in the different aspects of sociality. Meanwhile, a loss in any of the multiple aspects of sociality becomes irreversible, registering as an isolated event. The same is true of any gains in the multiple aspects. As a result, when it is necessary to calculate the status of sociality in total, discrete losses have to be balanced against discrete gains once and for all. Likewise, threats that the mask may bring to sociality are balanced against opportunities that the mask may produce simultaneously. Depending on the balance sheet, the mask becomes either an opportunity or a threat but not both. In this calculation, the mask touches on codified and essentialized aspects of sociality but not emergent ones.

Second, this static and limited view on the multiple aspects of sociality and the mask is reinforced when collectivists do not acknowledge the mediation of the individual elements of individuality for the dynamic reconstitution of these aspects of sociality which are in turn further foundations for individuality. Collectivists do not allow the correlativity and consubstantiality between individuality and sociality and, instead, consider the former reducible to the latter. Therefore, it is unthinkable that a population of corporeal bodies produce various non-bodily individualities like Simmel’s metropolitan individuals. These individuals in Simmel first exist as a population of blasé-faced corporeal bodies, which is the immediate foundation for corporeal individuality for them. Subsequently, Simmel observes that this population of bodies develops into various shapes of mentality (i.e., mental lives) from one individual to another. This correlative development of non-bodily individuality (i.e., an individual mind) from bodily individuality (i.e., an individual body) which is, in the beginning, derived consubstantially from bodily sociality (i.e., a population of bodies), can ultimately result in the development of new non-bodily sociality. For example, a supra-individual sense of bleakness or excitement about community (i.e., a kind of non-bodily sociality) can be generated by a simple trigger of one individual who does utter one’s emotion in terms of bleakness or excitement (i.e., a kind of non-bodily individuality) amid a multitude of masked faceless bodies (i.e., a kind of bodily sociality). Once heard, the initial individual utterance ripples across other individuals, resulting in a shared sense of a bleak or exciting instance of emotional sociality. Collectivists who dismiss individual elements apart from sociality do not admit this mediation of individual elements from one aspect of sociality to another. In other words, they do not acknowledge that sociality of a certain kind (e.g., a bodily one) can produce sociality of another kind (e.g., a non-bodily one) via the very mediation of individuality. Therefore, they downplay the possibility that sociality of a certain kind (e.g., non-bodily sociality) that is lost by the mask that simultaneously saves sociality of another kind (e.g., bodily sociality), can be regenerated by the latter through the mediation of individuality. As a result, the extent to which the mask intervenes in the making of sociality (i.e., the supra-individual foundation of individuality) becomes minimized to only a few pre-existing supra-individual elements.

Founded on this conception of the irreversibility and non-renewability of sociality, collectivists in empirical studies end up ascribing a limited set of meanings to the mask in considering whether it is a threat or an opportunity for sociality. First, studies report that social individuals wear the mask out of collectivist motivations to separate and save sociality from dangerous individuals. People in Hong Kong during the SARS outbreak in 2008 are described to act against “the possible extirpation of the social” by wearing the mask in a collectivist manner [101]. According to the study, Hong Kongers actively set up separating walls (i.e., face masks) around virus-transmitting individuals, “efface” facial distinctions among themselves, and instead produce collective resemblance and give salience to Hong Kong the social. Although it remains controversial whether Hong Kongers are concerned only with the extirpation of the social amid the SARS and not the possible extinction of the individual in their practicing the “efface work” of mask-wearing, the study suggests that the supra-individual entity of Hong Kong is taken as a critical foundation of Hong Kongers’ individuality. A similar observation is made among religious studies. According to the conservative Muslim understanding of female head-covering, hijabs are valid instruments to separate the external world (i.e., weak and unstable masculinity) from female bodies so that the former sustains itself without being endangered by the latter [79, 87: 75, 92]. The conservative understanding posits that the protected masculinity represents the whole world which is controversially the sole foundation of any feminine existence.

Second, the mask is a conduit through which material and spiritual powers from supra-individual entities are transposed into ego for the latter’s existence. Anthropological studies demonstrate that the mask indicates ancestors, animal spirits, and other supernatural powers [30, 111, 112]. While an emerging group of modern Chinese doctors propagated the bio-physical efficacy of the mask during the 1910–11 plague outbreaks in Manchuria, laypeople in Manchuria, on the other hand, are found to have worn the mask pressed with Buddhist temple stamps, engendering the mask a sort of mythic amulet which was deemed to be a supra-individual power granting good luck for survival [103]. Encountering these ordinary Manchurians, the modern Chinese doctors might have asserted their individuality as ‘scientific’ doctors by wearing the mask not only as a material barrier that disconnects them from another supra-individual power (i.e., unknown bio-material sources in the surrounding) [104: 46–47] but also a symbolic liaison to yet another supra-individual power (i.e., modern science vs. tradition and magic). What is revealed in common among laypeople and doctors is the symbolic power that the mask conveys from supra-individual entities onto individuals for the latter to assert for due individuality ultimately.

### The wall–conduit fold and the ego–world fold

The mask is ever more consequential for individuality than among atomists or collectivists to dualist social individuals who base agency and individuality on both individual and supra-individual entities. That is, firstly, because atomists are concerned with the mask’s impacts only upon individual entities for individuality, and collectivists are concerned with those only on supra-individual entities for individuality, whereas dualists are concerned with both. To the extent that dualists deal with the mask concerning both individual and supra-individual elements of individuality, they are likely to address the mask’s multiple and sometimes conflicting meanings.

Secondly, to the extent that dualists are more emergentist than essentialist, they ascribe complex meanings to the mask. While the essentialist perspective states that elements constitutive of individuality are pre-fixed and confined [113], the emergentist view acknowledges that these elements emerge in process expandingly [68, 90: 133–134, 114: 212] and transfusively [47: 89] across different aspects. Compared to atomists and collectivists, dualists are emergentist in that they appreciate the individual elements for individuality that originate contingently from the supra-individual elements; they acknowledge the supra-individual elements for individuality that frequently develop from the individual elements [68]. This emergentist and developmental perspective makes dualists prone to processes in which individuality reconstitutes itself in the unknown as well as pre-known manners.

Speaking of individuality based only on individual elements as among atomists, dualists are more susceptible than atomists to the dynamic process of renewing individuality in which individuality of a certain kind (e.g., bodily) can produce individuality of another kind (e.g., non-bodily) in novel ways. For example, the emergentist view acknowledges that non-bodily emotional existence is not constituted only by facial bodily existence but other emergent possibilities of bodily existence that are extra-facial, manual, and pedestrian when facial bodily existence is obstructed by the mask. When the mask threatens facial bodily existence, bodily existence evolves into the hand- and the foot-mediated corporeality which in turn only reconstitutes, but never forgoes, bodily existence which in turn reconstructs, but not forgoes, non-bodily emotional existence that used to be tied to the face only. Speaking of individuality reduced to supra-individual elements as among collectivists, dualists are likewise more susceptible than collectivists to the dynamic processes in which various aspects of sociality reconstitute one another in unforeseen manners.

For another elaboration, it is obvious that the mask wipes the face off individuals, denying facial bodily individuality and face-related non-bodily individuality such as emotion. In this sense, the mask produces the absence of individuality both in bodily and non-bodily aspects. What is present is only the outside of the masked person. In this process, however, the mask paradoxically indicates the presence of individuality inside the mask, being demarcated as a blank and a hole by the present surrounding. It is a sort of presence of absence, or absent presence [115]. While it is challenging to immediately specify what individuality the masked, absent presence refers to, the existence of this absent individuality is undeniably corporeal (if not facial) and real. By disguising an individual’s face in a noticeable and not in an invisible, stealthy manner [96: 96], mask-wearing ironically gives greater salience to individuality. That is because the mask covers the face and, by doing so, reveals the undefined and unspecified corporeal presence of individuality. Masked individuals experience the potentiality of another world and another identity by being this very kind of corporeal presence [89: 269]. Being undefined and unspecified, the masked individuality can be anything while being nothing in particular. The mask produces this anything-ness, by ostensibly separating and dividing its wearer from the surrounding, thus implicating the existence of something behind the mask, and yet simultaneously by blending and merging its wearer with the surrounding, thus implying nothing behind the mask other than the presence of the whole surrounding. This is how anonymity produces both identity negation and identity creation at once [97]. This ironic law of absent presence is the foundation for the unique power and awe that the mask wields over its wearers and bystanders in protests [89: 274–277, 109]. While masked protesters look anonymous, they deliver unfathomable possibilities and threats as political actors.

When the mask makes people (its wearers and bystanders alike) neglect individuality tied to the face, it prompts them to consider individuality in other terms that are non-facial or non-bodily [86–88, 116]. In other words, the mask succeeds in revealing powers and categories that are unknown and unspecified yet, by hiding boundaries and categories known so far [30]. This process of revealing by hiding maximizes the generative potential of the mask for the development of individuality that is often unattended on the existing terrains. Drawing on this emergent process, dualists are susceptible to multiple and often paradoxical effects of the mask on individuality which can sometimes be transformative enough to change its wearer from one category to another [103, 111].

Lastly, unlike atomists, dualists consider supra-individual elements relevant for the development of individual elements for agency and individuality (i.e., the mediation of supra-individual elements that help regenerate individual elements). While the mask saves one individual element and simultaneously threatens another element, this is not a once-and-for-all tradeoff on account of the mediation of supra-individual elements. For example, the mask can save an individual element for individuality (e.g., a corporeal body safeguarded under the mask) and simultaneously remove another individual element for individuality (e.g., emotion on the face hidden by the mask). The saved individual element by itself has no direct way to revive the lost individual element. The former rebuilds the latter, however, when the former develops into a supra-individual entity (e.g., a population of many corporeal bodies) in which many corporeal persons vie for developing distinct emotional individualities via fashion [61], such as designer masks and non-facial bodily performances, if not facial expressions.

Unlike collectivists, in addition, dualists acknowledge that sociality of a certain kind (e.g., a population of corporeal bodies) can produce sociality of another kind (e.g., a shared sense of community) via the mediation of individual entities (e.g., a corporeal body). A collective sense of community is often endangered by the mask that erases face-mediated affects (or communications) among individuals, while the same mask simultaneously saves a population of corporeal bodies. Yet, the former is rebuilt by the latter when the latter secures an individual body that further displays bodily affects (e.g., dancing or laboring), if not facial. These face-free, body-mediated individual affects ultimately develop one after another into foundations for the collective senses of community. When the mask influences one supra-individual element for individuality positively and another negatively, it is not a once-and-for-all tradeoff on account of this mediation of individual elements.

It is crucial to understand the consubstantiality between individuality and sociality in this process. In a phenomenological sense, entities and existence are real in “the harmony” and connection “between what we aim at and what is given, between intention and performance–and the body is our anchorage in a world” [32: 146]. This harmony in the body refers to the same two things. On one hand, if one asks whether one exists, this phenomenological formula answers that one certainly is in (relation to) the world as a body. On the other, if one asks whether the world is, it answers that the world certainly exists in (relation to) a person as the body foremost. For example, in markets and subway stations shunned by most people for fear of virus contraction in the early months of COVID-19, the number of masked individuals, though faceless, attests to the very extent to which sociality is physically present in the place. Every single body of masked individuals counts for sociality of such a bodily kind. To the extent to which individual bodies exist, society exists. In this sense, individuality and sociality are consubstantial. When individual bodies persist in physical places, carrying a symbol of sociality (e.g., wearing the government-sanctioned mask), sociality like a national community gains a lively, material footing in addition to its immaterial foundation [30, 112]. At the same time, once a population of masked bodies begins to form spatially, a hitherto secluded person may feel like presenting and asserting one’s body there. Sociality invites individuality this time. Once an individual body is connected to sociality in this way, the person is given an additional, supra-individual foundation for continuing to be oneself [30: 6, 112: 55]. Individuality is consubstantial with sociality.

On these accounts, dualists take multiple and conflicting meanings and voices from the mask more than atomists or collectivists. The mask both protects and threatens ego (or the world) at the same time; it both separates ego from the world and bridges ego to the world simultaneously; it both divides and combines ego and the world; it asserts one aspect of ego (or the world) over another, while it asserts yet another novel aspect of ego (or the world) during this process; it both asserts and denies ego (or the world) simultaneously; it anonymizes and identifies at once; it constrains its wearers to be bodily and thus enables them to be more than bodily. In this sense, the mask is a wall–conduit fold rather than a wall only or a conduit only. The mask is an ego–world fold rather than either ego or the world. The mask is concurrently for and against individuality and agency in various ways. In the mask do dualists observe ambivalence and multivocality.

### Hypothesis

The previous section highlights three points. First, the mask is consequential for individuality founded upon individual and supra-individual elements, acting upon individuality in three ways. The mask conceals and abnegates its inside (i.e., ego) and its outside (i.e., the world); it separates the inside from the outside, protecting them from each other; it reveals and asserts the inside and the outside to-and-fro. Second, these ways of the mask (i.e., concealment–abnegation, separation–protection, and revelation–assertion) are varyingly relevant to atomists, collectivists, and dualists. The ways in which the mask affects individuality concern atomists on individual elements (i.e., ego) and not supra-individual ones (i.e., the world); they concern collectivists on supra-individual and not individual elements; they concern dualists on both elements. To this extent, dualists ascribe more voices to the mask than atomists or collectivists. Third, in each unfolding of concealment–abnegation, separation–protection, and revelation–assertion, three types of social individuals take more or fewer meanings off the mask. Dualists ascribe multiple voices to the mask more than atomists or collectivists in the following elaboration. All three ways are universally identifiable across the types of social individuals.

Regarding the way of concealment–abnegation, the mask becomes an unequivocal threat to atomists and collectivists, whereas it becomes an incomplete, ambiguous threat to dualists. The mask covers up and abnegates pre-known individual elements (e.g., faces and emotions) and pre-known supra-individual elements (e.g., gatherings of faces). The former becomes a threat for individuality among atomists, while the latter becomes a threat for individuality among collectivists. At the same time, the mask accidentally exposes unknown individual elements (e.g., a face-covered body) while concealing the pre-known individual ones; it haphazardly reveals unknown supra-individual elements (e.g., gatherings of faceless bodies) while concealing the pre-known supra-individual ones. To this extent, the mask becomes ambivalent in constructing and deconstructing foundations for individuality. It is a vague, ambiguous threat to dualists who are susceptible to unknown, emergent individual and supra-individual elements for individuality. Atomists and collectivists are not so susceptible. In addition, the mask does not always conceal both individual and supra-individual elements simultaneously; while it conceals one (e.g., individual faces), it often reveals the other (e.g., governments that recommend or mandate the concealment). To dualists who are open to individual and supra-individual elements for individuality at once, therefore, the mask is an incomplete, ambiguous threat to individuality that is multi-faceted.

Regarding separation–protection, the mask becomes an unequivocal opportunity to atomists and collectivists while it becomes an incomplete, ambiguous opportunity to dualists. The mask separates and protects pre-known individual elements (e.g., an uninfected body) from the outside, guaranteeing individuality for atomists; the mask separates and protects pre-known supra-individual elements from capricious, unstable, and dangerous individual elements, ensuring individuality for collectivists. At the same time, the mask does not always protect individual and supra-individual elements from each other bilaterally; it sometimes protects only the former from the latter, while other times protecting only the latter from the former. To this extent, the mask is an ambiguous opportunity for individuality to dualists who base individuality on both sides. In addition, the mask accidentally discloses and endangers unknown individual elements to supra-individual elements while protecting the pre-known individual elements from the latter. On the other hand, it occasionally exposes and endangers unknown supra-individual elements to individual elements while protecting the pre-known supra-individual elements from the latter. To dualists whose individuality is founded upon unknown as well as known elements at both individual and supra-individual levels, therefore, the mask means an ambiguous opportunity that is multi-fold.

Regarding revelation–assertion, the mask means an unequivocal opportunity to atomists and collectivists, whereas it refers to an incomplete, ambiguous opportunity to dualists. The mask attests and asserts pre-known individual elements (e.g., a face-covered body that wears the mask) and pre-known supra-individual elements (e.g., governmental apparatuses that mandate the mask) by providing itself to them as the medium of action. The former means an opportunity for individuality among atomists, while the latter is for individuality among collectivists. At the same time, the mask does not simply assert one of the two elements; it often asserts one while simultaneously negating the other. Other times, it asserts both that are not necessarily in harmony and destructive of each other. To this extent, the mask is an ambiguous opportunity for individuality to dualists who place individuality on both individual and supra-individual elements. In addition, the mask contingently conceals unknown individual elements (e.g., varying facial expressions) while revealing pre-known individual elements (e.g., a face-covered body). It incidentally conceals unknown supra-individual elements (e.g., a shared sense of voluntary commitments to the demanding state) while asserting pre-known supra-individual elements (e.g., the demanding state). To dualists whose individuality is founded upon unknown and known elements, therefore, the mask means an ambiguous opportunity that is multivalent for their individuality.

HYPOTHESIS 2.—It is hypothesized that dualists ascribe more voices and meanings to the face mask against COVID-19 than atomists and collectivists do. Dualists are more likely to support multivalence and multivocality of the mask, as they perceive that the mask has to do with both ego and alters (rather than either side alone); all the alleged effects on emergent aspects of individuality, such as physical protection, psychological relief, and symbolic ritual (rather than only part of these); both life adversity and life betterment (rather than either one alone) that the mask brings to everyday life during the pandemic.

## Consequences of mask multivocality

So far, it is explicated that the mask has more or less multivocality among three different types of social individuals. It is a theoretical exercise to demonstrate that the mask that intervenes in the intersection of ego and the world varies in its signification from having simple to complex meanings. What implications does sociological theory get by discussing this varying multivocality in the mask? In a nutshell, the multivocality framework encourages us to understand and predict how people behave when they have to wear the mask not only in an emergency, like the current pandemic, but in everyday life as in the Goffmanesque sense [36, 37]. As a way to establish hypothetical patterns in the relationship between how people behave and how multivocal meanings they ascribe to the mask, the following elaborates two specific implications of mask multivocality. Several anthropological studies and political-sociological studies are especially relevant to the current conceptualization.

Multivocality implies both vulnerability and transformative power at once. These consequences derive from the fact that multivocality is both constituted by existing multiple meanings and constitutive of the unknown, novel meanings emerging from these existing meanings. First, people ascribe multiple meanings to the mask, as among dualists compared to atomists or collectivists, to the extent to which they already know these multiple meanings and boundaries upon which individual agency and individuality are founded so far. Existing studies elaborate that the multivocality of the mask mainly reflects the multiple existing boundaries that are incorporated in the mask [30, 38, 89, 109, 111, 117], such as new and old generations; successors and ancestors; life and death; presence and absence; safety and dangers; awe and terror; creation and destruction. Along these existential boundaries, people who read multiple meanings from the mask perceive impacts more readily that tumultuous times (or routines in everyday life) bring to their capacity to exist in the world. The more of the existential boundaries they draw on, the more susceptible they become to unfolding changes in life. Any adverse impacts will be perceived more sensitively by people ascribing more meanings to the mask. So are any positive impacts among people ascribing multiple meanings. When a social event generally brings about negative impacts along with positive ones, people who embed multiple meanings in the mask are more likely to appreciate them both. Furthermore, they are less likely to trade off these effects on any arbitrarily single plane. Instead, they likely accept the negativity on one dimension and the positivity on another concomitantly. Therefore, people who ascribe more meanings to the mask tend to be more vulnerable to any negative impacts and more susceptible to concurrent positive impacts.

In addition, once existing multiple boundaries are incorporated in the mask, people who take these boundaries off the mask deal with traversing the different and often contradictory boundaries by way of the very mask. To them, the mask becomes not only an intersection or a street corner [90: 133] where various boundaries come together but also a power with which they can potentially experience each of the many boundaries one after another. Studies attend to this process with the notion of transformative and communicative power [89, 109, 117] that the mask bestows to people confronting conventional boundaries. This view marks a significant theoretical advance that goes beyond the conventional view that the mask only separates, blocks, divides, and maintains various elements for individuality, such as the inside and the outside of the mask. The mask also connects, mixes, and bridges the inside and the outside. Therefore, people who read multiple meanings from the mask and even the paradoxes involved in it are more likely to accept that they maneuver through not only difficulties in the mask but opportunities by the mask.

Lastly, what the mask reveals is not only a transformative and communicative potential for individuals to move from a conventional boundary to another known one. The mask-mediated transformation and communication involve transformative experiences not only across multiple known categories, such as life and death, presence and absence, and the social and the individual, but also unknown emergent categories, such as life–death, present–absence (or absent–presence), blank–existence, and the social–individual. In encountering these hyphenated experiences, people fall short of identifying them with conventional boundaries and creatively generate new boundaries for them. These experiences hold such a transformative power that they do not preside simply in bridging existing boundaries but generating a new boundary that incorporates these existing ones altogether [cf. 68, 90]. In this sense, the mask conveys its uniquely generative power in its very multivocality, which is more significant in transformation than the bridging, transmissive power in the previous case. Therefore, people who appreciate the multivocality of the mask are likely to expect, while they are still in adversities, creative and generative futures [17] that are not simply opposites to existing adversities (i.e., good opportunities) but novel, transformative mixtures of adversities and opportunities at once. In this understanding of generative futures as mixtures of adversities and good opportunities, adversities are seen as nothing but bad opportunities that are as opportune as good ones as in a pragmatist perspective [70, 118]. On these grounds, a final set of hypotheses follow.

HYPOTHESIS 3–1.—As for the vulnerability side of mask multivocality, it is hypothesized that people who ascribe more meanings to the mask feel more vulnerable to damaging impacts of the COVID-19 pandemic and thus more willing to practice preventive measures like social distancing and feel the negative impacts of counter-COVID-19 measures.

HYPOTHESIS 3–2.—On account of the transformative power of the multivocal mask, people who ascribe more meanings to the mask are more likely to expect new and sustaining futures after the pandemic. While these people readily acknowledge future changes after the pandemic and their continuity, they expect new growth rather than catastrophic destruction in the future.

## Data and methods

To test three sets of hypotheses on the COVID-19 masquerade in S. Korea, this study uses the second wave data (administered for a week from Oct. 26 to Nov. 2, 2020; see Table 1 for details) from a web-based national panel survey of Koreans aged 20 or more, which comprises an international survey consortium of the Values in Crisis research [119]. This online panel is a quota sample (N = 4,000) originally constructed in May 2020 in proportion to the composition of the national population on three measures, such as age, gender, and place of residence. It is drawn from a Seoul-based research company’s (Embrain) online panel of 1.3 million voluntary participants. The number of respondents who have completed the second wave is 3,032 out of the original 4,000 invited to participate (response rate = 75.8%). The survey is approved by the IRB of Sungkyunkwan University and conducted under participants’ consent by way of participating in the survey online after reading the consent form. Maximum Likelihood Estimation (MLE) regression models for categorical dependent variables are specified via Stata [120].

**Table 1:** Descriptive statistics (N = 3032).

Variable		Frequency	Ratio	Variable		Frequency	Ratio
Types of Social Individuals				COVID-19 Symptoms			
	Nihilist	618	0.20		No	2924	0.96
	Atomist	428	0.14		Yes	108	0.04
	Collectivist	1013	0.34	COVID-19 Test			
	Dualist	973	0.32		No	2818	0.93
Multivocality of the Mask					Yes	214	0.07
	Mean 3.93 SD 0.49 Min 1 Max 5			COVID-19 Positive			
Gender					No	3011	0.99
	Male	1563	0.52		Yes	21	0.01
	Female	1469	0.48	Economic Hardship			
Age					No	2674	0.88
	20s	607	0.20		Yes	358	0.12
	30s	684	0.23	Collectivism			
	40s	855	0.38		Mean 4.18 SD 0.99 Min 1 Max 7		
	50s	620	0.20	Individualism			
	60s & more	266	0.09		Mean 2.44 SD 1.11 Min 1 Max 5		
Place of Residence				Locus of Control (LOC)			
	Metropolis	1425	0.47		Mean 2.67 SD 0.55 Min 1 Max 5		
	Suburb	648	0.21	Social Distancing			
	Small City	816	0.27		Not Very Much	32	0.01
	Rural Area	144	0.05		Well	270	0.09
Marital Status					Somewhat	1919	0.63
	Single	1212	0.40		Very Much	811	0.27
	Married	1702	0.56	Sense of Social Isolation			
	Wid/Div/Sep	118	0.04		Never	281	0.09
Education					Almost Never	682	0.23
	High School or Less	590	0.19		Occasionally	1342	0.44
	College or More	2442	0.81		Sometimes	541	0.18
Income (Household, Monthly)^1)^					Always	186	0.06
	Less than 1 M Won	258	0.09	Post-COVID-19 Change			
	1~3 M Won	985	0.32		Damage	735	0.24
	3~5 M Won	905	0.30		No Change	1114	0.37
	5~8 M Won	647	0.21		Growth	1183	0.39
	More than 8 M Won	237	0.08	Social Distancing in Place			
Religion					Don’t Agree at All	91	0.03
	None	1449	0.48		Don’t Agree	309	0.10
	Protestant	678	0.22		Well	876	0.29
	Buddhist	486	0.16		Agree	1420	0.47
	Catholic	328	0.11		Agree Very Much	336	0.11
	Other	91	0.03	Natural Conservation in Place			
Political Liberalism					Don’t Agree at All	227	0.08
	Mean 5.92 SD 1.85 Min 1 Max 10				Don’t Agree	800	0.26
Worry of COVID-19					Well	1239	0.41
	Not Worried	382	0.13		Agree	619	0.20
	Worried a Little	1919	0.63		Agree Very Much	147	0.05
	Worried a Lot	731	0.24				
Perceived Likelihood of COVID-19 Infection							
	Not Likely	826	0.27				
	Somewhat Likely	1983	0.65				
	Very Likely	223	0.08				

^1)^ One million wons approximate USD 800.

### Key variables

*Types of social individuals* are measured by whether respondents agree on a set of two statements tailored to the current pandemic experiences: “during the COVID-19 pandemic, it is impossible to secure public health security unless we protect individual rights;” “during the COVID-19 pandemic, it is acceptable to restrict individual rights for the sake of public health security.” Respondents who agree on the first statement and not on the second are operationalized as people who regard individual elements rather than supra-individual ones for their existence and agency amid the pandemic (*individualist* social individuals). Those who agree on the second and not on the first are people who regard supra-individual elements over individual ones for their existence (*collectivist* social individuals). People who agree on both are those who regard both individual and supra-individual elements at once for their existence (*dualist* social individuals). In the sample, the proportions of three types of social individuals are 14% (atomists), 34% (collectivists), and 32% (dualists). Dualists are as many as collectivists, while atomists are the least in Korea. Respondents who do not agree on either are named *nihilists* who do not reveal concerns about their existence in terms of these two statements (20% in the sample). Nihilists do not exist in the typology of social individuals. If any, they are those who do not consider one’s own capacity to engage with the world with various individual and supra-individual elements, which runs counter to the fundamental premise in action theory that human beings become subjects with agency and individuality. To the extent to which action theory and the typology of social individuals exhaust the possible ways of social individuals, the theoretical model presumes no nihilists. Therefore, one way to reconcile the theoretical nonexistence with the handful existence of respondents improvisationally named nihilists in the data lies in resolving the limitation of the two questionnaire items that are used to operationalize the three types of social individuals. The two questionnaire items may have missed some individuals who construct their agency and individuality as atomists, collectivists, and dualists in other terms than the two. One may need to develop a more comprehensive set of questionnaire items to this end.

*Multivocality of the mask*, defined as the extent to which people ascribe multiple meanings and voices to the face mask, is measured by a composite variable that takes the average of responses to how much a respondent agrees on each of the following seven statements (i.e., the average response to seven questions): the face mask is for alters; it is for ego; it protects from contracting COVID-19; it provides a psychological relief; it signals mutual regards among people; it makes my everyday life difficult; it makes my everyday life better. Responses to each are measured from 1 (disagree) to 5 (agree). The higher the average is, the more meanings people take from the face mask.

### Antecedents

For these two key variables, the first set of hypotheses test whether the proposed types of social individuals are reasonable enough to correspond with existing measures, such as collectivism (Hypothesis 1–1), individualism (Hypothesis 1–2), and the locus of control in life (LOC) (Hypothesis 1–3). *Collectivism* [78] is measured by the average of responses (1, disagree to 7, agree) to how much a respondent agrees that “it is not right to oppose to what most other people accept, even if it contradicts what I think right” and that “it is not right to complain about others if I want to get along with them.” *Individualism* [78] is measured by responses to a single item asking whether it is government or individual responsibility to reduce income gaps and inequality in the country (1, government responsibility to 5, individual responsibility). The mean of collectivism is 4.18, while individualism is averaged at 2.44. These means suggest that collectivism is more prevalent than individualism in Korea. *The locus of control* is measured by a composite variable that takes the average of responses (1, disagree to 5, agree) to how much a respondent agrees on each of the following six statements: my life is determined by my own action; by powerful others; by accidental happenings and luck; by God; by ancestors; by natural environments. This study added two elements (i.e., ancestors and natural environments) to the original list of four in the literature on the locus of control in life [79, 80]. The higher the value is, the more loci the respondent places control powers over one’s life in.

Hypothesis 2 tests whether mask multivocality is associated with the types of social individuals. Bivariate analysis shows that atomists score 3.83 on average for mask multivocality; collectivists, 3.98; dualists, 4.11. The complete treatment of this hypothesis follows in the results section.

### Consequences

Once the relationship between social individuals and mask multivocality is specified, the last set of hypotheses investigates what behavioral and attitudinal consequences mask multivocality predicts. Five variables are used to measure some consequences. *Social distancing* refers to the extent to which a respondent complies with government measures of social distancing (e.g., restrictions on socializing, shopping, schools, and workplaces). Responses are measured by 1 (not very much), 2 (well), 3 (somewhat), and 4 (very much). Most respondents comply with social distancing measures somewhat (67%) or very much (23%), while 10% remain weak on their compliance. *Sense of social isolation* refers to how often a respondent feels that one does not have enough socializing in the past four weeks (1, never to 5, very often). These two variables are used to test Hypothesis 3–1.

Regarding Hypothesis 3–2, three variables are built. *Post-COVID-19 change* measures whether a respondent thinks that one’s country will grow a lot or get damaged a lot after COVID-19. Responses are coded ‘damage’ (24%), ‘no change’ (37%), and ‘growth’ (39%). *Social distancing in place* measures responses (1, disagree to 5, agree) to how much a respondent agrees that social distancing practices (e.g., work-at-home and restricted socializing) will be in place after the pandemic is over. *Natural conservation in place* measures responses (1, disagree to 5, agree) to how much a respondent agrees that people will prioritize natural conservation over economic exploitation after the pandemic.

### Confounders

A set of potential confounders are controlled, such as socio-economic statuses like age, gender, place of residence, marital status, education, income, religion, and political liberalism. Religion and political liberalism are included as religious and political orientations are often argued to confound the relationships among the focal variables in the following analyses [121, 122]. In addition, experiences of the pandemic are considered in the models. *Worry of COVID-19* measures how worried a respondent is about getting infected; *Perceived likelihood of COVID-19* measures how likely a respondent thinks one may get infected; *COVID-19 symptoms* measure whether a respondent has ever had light or severe symptoms of COVID-19. *COVID-19 test* measures whether a respondent has ever tested for the COVID-19 infection. *COVID-19 positive* measures whether a respondent has ever tested positive; *Economic hardship* measures whether a respondent has ever lost one’s job, become a temporary employee, or closed one’s business due to the pandemic. The impacts of income on social practices during the pandemic are often found to be different from those of the economic hardship induced by the pandemic [123, 124].

## Results

### Who are atomists, collectivists, and dualists?

To elaborate who are atomist, collectivist, and dualist social individuals, Table 2 reports three regression models to test three hypotheses: Model 1 for Hypothesis 1–1 on how collectivism leads to the types of social individuals, Model 2 for Hypothesis 1–2 on the effect of individualism on the types, and Model 3 for Hypothesis 1–3 on the effect of the locus of control. Since the dependent variable (i.e., type of social individuals) is a nominal variable involving four response categories, coefficients are reported as relative rate ratios from multinomial logistic regression models. The base category is set at ‘dualist.’

**Table 2:** Relative rate ratios of becoming different types of social individuals (atomists and collectivists vs. dualists) from multinomial logistic regression models.

	Model1			Model2			Model3		
	Nihilist	Atomist	Collectivist	Nihilist	Atomist	Collectivist	Nihilist	Atomist	Collectivist
Collectivism	0.79**	0.74**	0.99						
	(0.043)	(0.045)	(0.047)						
Individualism				1.00	1.05	0.89**			
				(0.048)	(0.055)	(0.038)			
Locus of Control							0.69**	0.53**	0.54**
							(0.067)	(0.059)	(0.047)
Gender (ref: male)	0.72**	0.89	0.82*	0.72**	0.90	0.81*	0.71**	0.87	0.79*
	(0.077)	(0.107)	(0.076)	(0.077)	(0.108)	(0.075)	(0.076)	(0.106)	(0.074)
Age (ref: 20s)									
30s	0.85	1.21	1.36*	0.84	1.19	1.36*	0.83	1.17	1.33+
	(0.142)	(0.243)	(0.205)	(0.140)	(0.239)	(0.206)	(0.139)	(0.235)	(0.203)
40s	0.77	1.14	1.05	0.76	1.10	1.05	0.74+	1.07	1.01
	(0.139)	(0.244)	(0.171)	(0.135)	(0.233)	(0.171)	(0.133)	(0.228)	(0.166)
50s	0.90	1.24	1.20	0.85	1.15	1.19	0.84	1.11	1.17
	(0.181)	(0.291)	(0.213)	(0.168)	(0.267)	(0.211)	(0.167)	(0.261)	(0.210)
60s or More	0.95	1.51	1.57*	0.85	1.32	1.56*	0.84	1.29	1.52+
	(0.245)	(0.434)	(0.353)	(0.218)	(0.374)	(0.351)	(0.215)	(0.368)	(0.344)
Place of Residence (ref: metropolis)									
Suburb	0.97	0.91	0.91	0.97	0.91	0.90	0.96	0.91	0.90
	(0.133)	(0.141)	(0.109)	(0.132)	(0.141)	(0.108)	(0.132)	(0.141)	(0.109)
Small City	1.01	0.86	1.01	1.02	0.87	1.01	1.01	0.86	1.00
	(0.129)	(0.126)	(0.112)	(0.130)	(0.127)	(0.112)	(0.129)	(0.127)	(0.111)
Rural	1.23	1.27	0.94	1.24	1.29	0.92	1.20	1.22	0.89
	(0.309)	(0.346)	(0.219)	(0.311)	(0.351)	(0.216)	(0.302)	(0.333)	(0.210)
Marital Status (ref: single)									
Married	0.79	1.04	0.77*	0.78+	1.03	0.78+	0.77+	1.01	0.75*
	(0.116)	(0.176)	(0.097)	(0.114)	(0.171)	(0.099)	(0.113)	(0.170)	(0.097)
Wid/Div/Sep	0.74	1.09	1.00	0.72	1.07	0.99	0.69	0.98	0.94
	(0.233)	(0.368)	(0.255)	(0.225)	(0.359)	(0.252)	(0.217)	(0.332)	(0.241)
Education (ref: high school or less)	0.93	1.04	1.13	0.95	1.08	1.10	0.96	1.09	1.15
	(0.124)	(0.160)	(0.135)	(0.126)	(0.165)	(0.133)	(0.128)	(0.167)	(0.140)
Income (ref: less than 1M won)									
1-3M Won	0.84	0.92	1.08	0.83	0.89	1.08	0.83	0.90	1.09
	(0.164)	(0.210)	(0.202)	(0.160)	(0.201)	(0.202)	(0.162)	(0.206)	(0.206)
3-5M Won	0.83	0.81	1.14	0.83	0.79	1.14	0.82	0.80	1.13
	(0.169)	(0.192)	(0.218)	(0.166)	(0.186)	(0.219)	(0.166)	(0.189)	(0.219)
5-8M Won	0.89	0.93	1.47+	0.90	0.92	1.47+	0.89	0.91	1.44+
	(0.196)	(0.235)	(0.297)	(0.196)	(0.231)	(0.299)	(0.195)	(0.231)	(0.295)
More Than 8M Won	0.52*	0.89	1.09	0.52*	0.86	1.11	0.51*	0.87	1.07
	(0.146)	(0.262)	(0.259)	(0.144)	(0.253)	(0.266)	(0.142)	(0.255)	(0.257)
Religion (ref: none)									
Protestant	0.87	0.95	0.89	0.86	0.93	0.89	0.93	1.07	1.01
	(0.118)	(0.144)	(0.106)	(0.117)	(0.141)	(0.107)	(0.128)	(0.164)	(0.123)
Buddhist	0.70*	0.82	0.87	0.71*	0.84	0.87	0.74+	0.89	0.92
	(0.111)	(0.141)	(0.115)	(0.113)	(0.143)	(0.115)	(0.117)	(0.153)	(0.123)
Catholic	0.92	0.82	1.18	0.93	0.84	1.18	0.99	0.92	1.30+
	(0.170)	(0.174)	(0.180)	(0.171)	(0.176)	(0.181)	(0.182)	(0.196)	(0.201)
Other	1.21	1.11	1.08	1.20	1.09	1.08	1.23	1.14	1.11
	(0.366)	(0.392)	(0.301)	(0.362)	(0.385)	(0.302)	(0.370)	(0.404)	(0.314)
Political Liberalism	0.92**	0.82**	1.03	0.91**	0.82**	1.02	0.91**	0.81**	1.02
	(0.027)	(0.027)	(0.026)	(0.027)	(0.027)	(0.026)	(0.027)	(0.027)	(0.026)
Worry of Covid-19 (ref: not worried)									
Somewhat Worried	0.73+	0.75	1.05	0.70*	0.71+	1.05	0.70*	0.72+	1.06
	(0.122)	(0.143)	(0.164)	(0.116)	(0.134)	(0.164)	(0.117)	(0.137)	(0.167)
Worried a Lot	0.55**	0.70	0.73+	0.52**	0.64*	0.73+	0.53**	0.68+	0.77
	(0.112)	(0.159)	(0.134)	(0.103)	(0.143)	(0.134)	(0.107)	(0.154)	(0.142)
Perceived Likelihood of Covid-19 Infection (ref: not likely)									
Somewhat Likely	0.99	1.04	1.11	1.02	1.10	1.10	1.04	1.13	1.14
	(0.128)	(0.153)	(0.124)	(0.132)	(0.160)	(0.123)	(0.134)	(0.166)	(0.129)
Very Likely	0.88	0.83	0.53**	0.94	0.90	0.54**	0.97	0.95	0.57*
	(0.206)	(0.219)	(0.120)	(0.217)	(0.236)	(0.121)	(0.227)	(0.250)	(0.129)
Covid-19 Test (ref: no)	1.67*	1.13	1.11	1.67**	1.13	1.13	1.71**	1.18	1.15
	(0.330)	(0.269)	(0.210)	(0.330)	(0.269)	(0.212)	(0.340)	(0.282)	(0.219)
Economic Hardship (ref: no)	1.12	1.18	0.99	1.12	1.18	1.00	1.16	1.25	1.05
	(0.182)	(0.215)	(0.148)	(0.182)	(0.214)	(0.148)	(0.189)	(0.228)	(0.157)
Constant	8.33**	6.24**	0.83	3.23**	1.71	1.11	9.02**	10.63**	4.23**
	(3.198)	(2.718)	(0.298)	(1.115)	(0.677)	(0.358)	(3.740)	(4.999)	(1.617)
Observations	3,032	3,032	3,032	3,032	3,032	3,032	3,032	3,032	3,032

Exponentiated standard errors in parentheses

** p<0.01

* p<0.05

+ p<0.1

**Fig 1:**
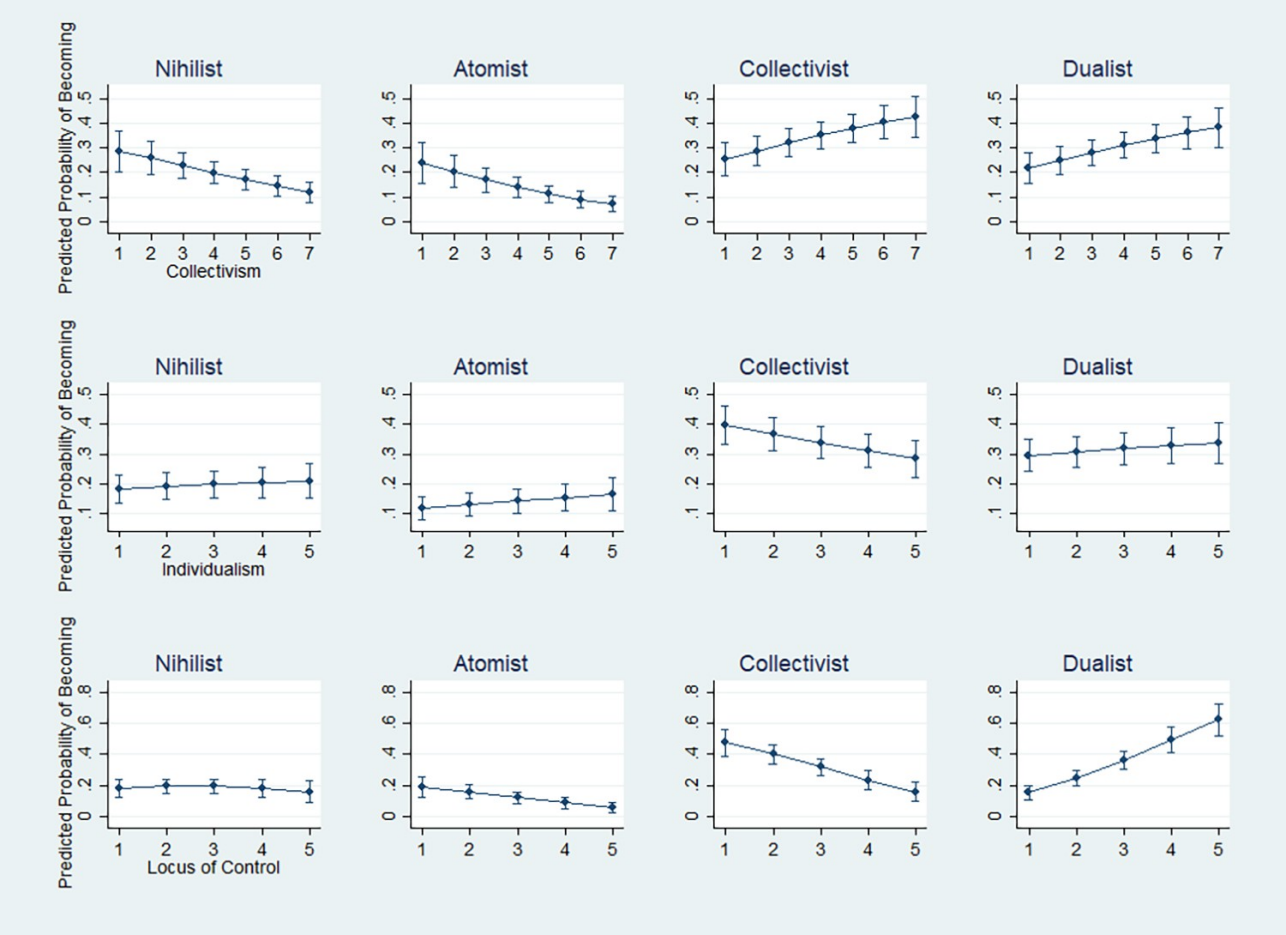
Changing probabilities of becoming atomists, collectivists, and dualists, depending on collectivism, individualism, and locus of control. Probabilities (dots with 95% CI ticks) are predicted for a person who holds modal values in gender (male), age (40s), residence (a metropolitan area), marital status (married), education (college or more), religion (non-religionist), household income (3-5M won), political liberalism (6 out of 10), COVID-19 worry (worried a little), COVID-19 likelihood (a little likely), COVID-19 test (no), and economic hardship (no).

Model 1 shows that the effect of collectivism is statistically significant for the probability of becoming atomists versus dualists. In detail, compared to the probability of becoming dualists, that of becoming atomists decreases by 26% (= 1–0.74) when collectivism increases by one unit. As respondents show more collectivism, they are less likely to be atomists than dualists. On the other hand, the effect of collectivism upon becoming collectivists versus dualists is insignificant. As collectivism increases, the probability of becoming collectivists and that of becoming dualists change in an indistinguishable way. The top row of graphs in Fig 1 depicts this pattern more intuitively. The Y-axis refers to the probability of becoming nihilists, atomists, collectivists, or dualists. The X-axis refers to changing values in collectivism from 1 to 7. As collectivism increases, the probability of becoming nihilists or atomists decreases, while the probability of becoming collectivists or dualists increases. This result supports Hypothesis 1–1 and shows that the proposed conceptual types of social individuals hold true against a common measure of collectivism. It also indicates that dualists are indistinguishable from collectivists in terms of collectivism; dualists are as collectivist as collectivists are.

Model 2 of Table 2 shows the effect of individualism. As individualism increases by one unit, the probability of becoming collectivists versus dualists decreases by 11%, while the probability of becoming atomists versus dualists does not change significantly. Graphically, the middle row of Fig 1 shows that individualism increases the chance of becoming atomists or dualists, while it decreases the probability of becoming collectivists. This result supports Hypothesis 1–2 and the theoretical types of social individuals. It also shows that dualists are indistinguishable from atomists in terms of individualism; dualists hold as individualist attitudes as atomists do. A critical pattern emerges from these top and middle rows of Fig 1. Koreans are more likely to be dualists as they become either more individualist or collectivist. That is to say, dualists are both individualist and collectivist at once, which is consistent with the conceptual typification of dualist social individuals in the theory part.

Model 3 of Table 1 further elaborates the characteristics of each type of social individuals by showing the effect of the number of the loci of control in life. The greater the number of control loci (i.e., the average of the locus of control variable) is, the more diverse locations people confirm the presence of controlling powers for their life at (i.e., ego, strong others, accidental happenings, God, ancestors, and nature). As the locus of control increases by one unit, the probability of becoming atomists and collectivists (versus dualists) decreases by 47% and 46%, respectively. In the bottom row of Fig 1, this pattern becomes clear in predicted probabilities. Koreans are less likely to become atomists or collectivists and yet more likely to be dualists, as their locus of control increases in number. The unique characteristic of dualists is highlighted in this pattern. Dualists find their powers for controlling and mastering their life in more diverse locations than atomists or collectivists do. This result supports Hypothesis 1–3.

In a further visualization of the result (not reported in this paper but available from the author upon request), factors that boost the average of the locus of control for atomists are found to be ego alone, while the other factors dampen it among atomists. Boosting factors for collectivists are powerful others and accidental happenings, while the others are suppressing. Factors increasing the locus of control for dualists are all the six elements, including God, ancestors, and natural environments, in addition to those for collectivists. Thus, compared to collectivists, dualists are more religious, spiritual, or naturalist; all the while, dualists are as much oriented to powerful others and accidental happenings as collectivists are. Compared to atomists, dualists are more extra-egoist in all aspects; all the while, dualists are as egoist as atomists are. In what follows, this study examines how these different social individuals signify the face mask in the current pandemic.

### Social individuals and mask multivocality

In a schematic interpretation of the sociology and anthropology of the mask in this study, voices and meanings of the mask have been specified into seven to develop survey questionnaire items: ego, alters, physical protection, psychological relief, a symbolic signal for mutual respect, life adversity, and life betterment. This section on Hypothesis 2 reports how many of these voices different types of social individuals ascribe to the COVID-19 face mask, as a way to show that multiple and contradictory meanings surrounding the face mask in Korea are related to the fact that Koreans are social individuals of various shapes. Since the dependent variable, mask multivocality, is a continuous variable, the analysis specifies the relationship between mask multivocality and social individuals with Ordinary Least Squared (OLS) regression models (Table 3).

**Table 3:** Unstandardized regression coefficients from OLS models of the number of meanings of the mask regressed upon the type of social individuals and other covariates.

	Model1	Model2	Model3	Model4	Model5	Model6	Model7	Model8
Types of Social Individuals (ref: dualist)								
Nihilist	-0.46**	-0.44**	-0.43**	-0.43**	-0.44**	-0.44**	-0.43**	-0.42**
	(0.024)	(0.023)	(0.023)	(0.023)	(0.024)	(0.024)	(0.023)	(0.023)
Atomist	-0.28**	-0.26**	-0.25**	-0.26**	-0.26**	-0.26**	-0.26**	-0.26**
	(0.027)	(0.026)	(0.026)	(0.026)	(0.027)	(0.026)	(0.026)	(0.026)
Collectivist	-0.13**	-0.13**	-0.12**	-0.12**	-0.13**	-0.13**	-0.13**	-0.12**
	(0.021)	(0.020)	(0.020)	(0.020)	(0.020)	(0.020)	(0.020)	(0.020)
Gender (ref: male)		0.19**	0.18**	0.19**	0.19**	0.19**	0.18**	0.18**
		(0.017)	(0.017)	(0.017)	(0.017)	(0.017)	(0.017)	(0.017)
Age (ref: 20s)								
30s		0.03	0.03	0.03	0.03	0.03	0.03	0.03
		(0.027)	(0.027)	(0.027)	(0.027)	(0.027)	(0.027)	(0.027)
40s		-0.04	-0.03	-0.04	-0.04	-0.04	-0.05	-0.03
		(0.029)	(0.029)	(0.029)	(0.029)	(0.029)	(0.029)	(0.029)
50s		-0.05	-0.04	-0.04	-0.05	-0.05	-0.05	-0.04
		(0.032)	(0.032)	(0.032)	(0.032)	(0.032)	(0.032)	(0.032)
60s or More		-0.09*	-0.08*	-0.08*	-0.09*	-0.09*	-0.09*	-0.08+
		(0.040)	(0.040)	(0.040)	(0.040)	(0.040)	(0.040)	(0.040)
Place of Residence (ref: metropolis)								
Suburb		0.01	0.02	0.01	0.01	0.01	0.01	0.02
		(0.022)	(0.021)	(0.022)	(0.022)	(0.022)	(0.022)	(0.021)
Small City		0.00	0.01	0.00	0.00	0.00	0.00	0.01
		(0.020)	(0.020)	(0.020)	(0.020)	(0.020)	(0.020)	(0.020)
Rural		0.00	0.02	0.01	0.00	0.01	0.00	0.02
		(0.040)	(0.040)	(0.040)	(0.040)	(0.040)	(0.040)	(0.040)
Marital Status (ref: single)								
Married		0.03	0.02	0.03	0.03	0.03	0.03	0.02
		(0.023)	(0.023)	(0.023)	(0.023)	(0.023)	(0.023)	(0.023)
Wid/Div/Sep		-0.06	-0.06	-0.06	-0.06	-0.07	-0.06	-0.06
		(0.047)	(0.047)	(0.047)	(0.047)	(0.047)	(0.047)	(0.046)
Education (ref: high school or less)		-0.00	-0.00	0.00	-0.00	-0.00	0.00	0.00
		(0.021)	(0.021)	(0.021)	(0.021)	(0.021)	(0.021)	(0.021)
Income (ref: less than 1M won)								
1-3M Won		0.01	0.01	0.00	0.01	0.01	0.01	0.01
		(0.032)	(0.032)	(0.032)	(0.032)	(0.032)	(0.032)	(0.032)
3-5M Won		0.03	0.03	0.02	0.03	0.03	0.03	0.03
		(0.033)	(0.033)	(0.033)	(0.033)	(0.033)	(0.033)	(0.033)
5-8M Won		0.02	0.02	0.01	0.02	0.02	0.02	0.02
		(0.035)	(0.035)	(0.035)	(0.035)	(0.035)	(0.035)	(0.035)
More Than 8M Won		0.01	0.02	0.01	0.01	0.01	0.02	0.02
		(0.042)	(0.042)	(0.042)	(0.042)	(0.042)	(0.042)	(0.042)
Religion (ref: none)								
Protestant		0.02	0.02	0.01	0.02	0.01	0.02	0.01
		(0.021)	(0.021)	(0.021)	(0.021)	(0.021)	(0.021)	(0.021)
Buddhist		-0.01	-0.02	-0.02	-0.01	-0.01	-0.01	-0.02
		(0.024)	(0.024)	(0.024)	(0.024)	(0.024)	(0.024)	(0.024)
Catholic		-0.02	-0.03	-0.02	-0.02	-0.02	-0.02	-0.03
		(0.028)	(0.028)	(0.028)	(0.028)	(0.028)	(0.028)	(0.028)
Other		-0.05	-0.05	-0.05	-0.05	-0.05	-0.04	-0.05
		(0.049)	(0.049)	(0.049)	(0.049)	(0.049)	(0.049)	(0.049)
Political Liberalism		0.01**	0.01**	0.01**	0.01**	0.01**	0.02**	0.02**
		(0.005)	(0.005)	(0.005)	(0.005)	(0.005)	(0.005)	(0.005)
Worry of COVID-19 (ref: not worried)								
Somewhat Worried			0.07**					0.07*
			(0.025)					(0.027)
Worried a Lot			0.18**					0.16**
			(0.029)					(0.032)
Perceived Likelihood of COVID-19 Infection (ref: not likely)								
Somewhat Likely				0.03+				0.00
				(0.019)				(0.020)
Very Likely				0.13**				0.04
				(0.035)				(0.038)
COVID-19 Symptoms (ref: no)					0.03			0.04
					(0.045)			(0.046)
COVID-19 Test (ref: no)						0.03		0.05
						(0.032)		(0.034)
COVID-19 Positive (ref: no)							-0.34**	-0.42**
							(0.100)	(0.105)
Constant	4.11**	3.92**	3.82**	3.88**	3.92**	3.92**	3.92**	3.81**
	(0.015)	(0.048)	(0.053)	(0.050)	(0.049)	(0.049)	(0.048)	(0.053)
Observations	3,032	3,032	3,032	3,032	3,032	3,032	3,032	3,032
R-squared	0.116	0.164	0.176	0.168	0.164	0.164	0.167	0.181

Standard errors in parentheses

** p<0.01

* p<0.05

+ p<0.1

Model 1 shows that dualists, as the reference group, score the highest on mask multivocality. Collectivists and atomists score less than dualists by 0.13 and 0.28, respectively. This pattern stays robust in the remaining Models 2 to 8. Models 2 to 7 incorporate measures of COVID-19 experiences as potential confounders one by one; Model 8 incorporates them all. Across all the models, dualists ascribe more meanings to the mask than collectivists or atomists, supporting Hypothesis 2 unequivocally. Dualists who place agency and individuality on both individual and supra-individual elements tend to take more meanings from the face mask than atomists or collectivists.

**Fig 2:**
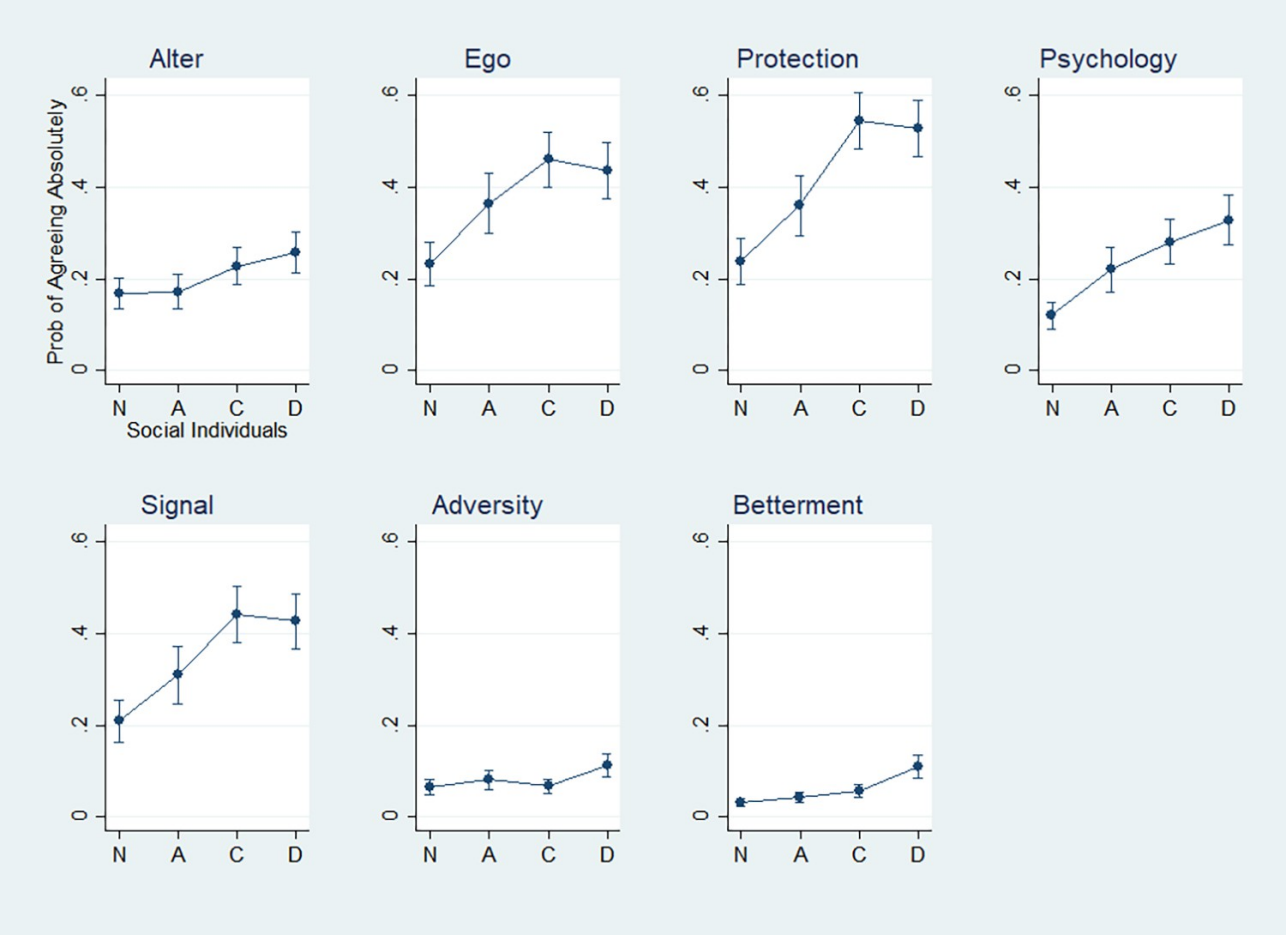
Predicted probabilities of agreeing absolutely on the meaning of the mask (“face mask is for (the stated meaning)”) by the type of social individuals (N: Nihilists; A: Atomists; D: Collectivists; D: Dualists). Probabilities (dots with 95% CI ticks) are predicted for a person who holds modal values in gender (male), age (40s), residence (a metropolitan area), marital status (married), education (college or more), religion (non-religionist), household income (3-5M won), political liberalism (6 out of 10), COVID-19 worry (worried a little), COVID-19 likelihood (a little likely), COVID-19 symptoms (no), COVID-19 test (no), and COVID-19 positive (no).

In order to elaborate which meanings different social individuals take from the mask one by one, this study has conducted a supplementary analysis of ordered logistic regression models (available from the author upon request) in which the extent to which a respondent agrees on each of the seven meanings of the mask is regressed upon the type of social individuals with an identical set of covariates as in Model 8 of Table 3. For ease of interpretation, this study has generated from the supplementary analysis predicted probabilities of agreeing absolutely on each of the seven meanings (Fig 2). It reveals two points. First, dualists agree the most strongly on all seven meanings (leading in alter, psychology, adversity, and betterment), followed by collectivists (leading in ego, protection, and signal). The extent to which Koreans agree on the stated meanings of the mask is the smallest among atomists. In detail, the meanings of ego, protection, and a signal of mutual respect emerge clearly among collectivists and dualists alike. The meanings of alters, psychology, life adversity, and life betterment are recognized more among dualists than collectivists. While acknowledging fewer meanings, atomists take ego and protection more frequently from the mask than the other meanings. In terms of the sequence of the most apparent meanings within each type of social individuals, there is little difference among atomists, collectivists, and dualists. The dominant sequence is protection, ego, signal, psychology, alters, adversity, and betterment. A cross-type difference instead exists in the extent to which social individuals take each of these meanings.

Second, another cross-type difference is in the extent to which social individuals take from the mask seemingly contradictory or parallel meanings simultaneously. This tendency is the greatest among dualists. In detail, while all three types of social individuals take ego and alters as the mask’s voice (the former more than the latter), the gap between the probability of agreeing absolutely on ego and that for alters becomes the smallest among dualists. The same is true of the two meanings of physical protection and psychological relief. Dualists take both meanings more simultaneously than atomists or collectivists. It is also true of the two contradictory meanings of the mask as life adversity and life betterment. While atomists and collectivists take adversity more than betterment, dualists take them both at the same level. In sum, these results support that different social individuals take different sets of meanings from the mask and that dualist social individuals take the most meanings from the mask that are contradictory or parallel with one another.

### Mask multivocality and the pandemic practices

It is proposed in the theory section that mask multivocality implies both vulnerability and transformative power at once. As for the former, Hypothesis 3–1 states that people who take more meanings from the mask are more vulnerable individuals so that they are more likely to abide by social distancing measures and feel negative impacts of counter-COVID-19 measures. For the latter, Hypothesis 3–2 states that people who take more meanings from the mask are more transformative individuals so that they are more likely to anticipate future changes after the pandemic and their continuity; they expect new growth rather than catastrophic destruction in the aftermath of the pandemic.

Table 4 addresses Hypothesis 3–1. Models 1 to 3 use social distancing as the dependent variable and show that mask multivocality leads to more compliance with social distancing measures imposed by central and local governments. This pattern is robust across the models. Models 4 to 6 use the sense of social isolation as the dependent variable and show that mask multivocality leads to a greater sense of social isolation during the pandemic. These results are supportive of Hypothesis 3–1. Additional analyses using the same models with, alternatively, the OLS estimation and the generalized ordered logistic regression method produce the same results (available from the author upon request).

**Table 4:** Odds ratios from the ordered logistic models of social distancing (models 1 to 3) and the sense of social isolation (models 4 to 6) regressed upon the multivocality of the mask and other covariates.

	Model1	Model2	Model3	Model4	Model5	Model6
	DV: Social Distancing			DV: Sense of Social Isolation		
Multivocality of the Mask	3.64**	3.77**	3.63**	1.21**	1.27**	1.20*
	(0.295)	(0.316)	(0.310)	(0.083)	(0.090)	(0.086)
Gender (ref: male)		1.28**	1.23**		0.90	0.90
		(0.100)	(0.098)		(0.062)	(0.063)
Age (ref: 20s)						
30s		0.73*	0.69**		1.14	1.15
		(0.091)	(0.087)		(0.126)	(0.127)
40s		0.96	0.94		1.19	1.25+
		(0.129)	(0.127)		(0.141)	(0.149)
50s		1.49**	1.42*		1.16	1.22
		(0.219)	(0.211)		(0.150)	(0.159)
60s or More		2.56**	2.25**		1.11	1.21
		(0.467)	(0.416)		(0.180)	(0.197)
Place of Residence (ref: metropolis)						
Suburb		0.86	0.88		0.97	0.98
		(0.085)	(0.089)		(0.085)	(0.086)
Small City		0.75**	0.75**		0.87+	0.88
		(0.069)	(0.070)		(0.071)	(0.072)
Rural		0.78	0.76		0.67*	0.70*
		(0.145)	(0.143)		(0.111)	(0.115)
Marital Status (ref: single)						
Married		1.08	1.09		0.87	0.83*
		(0.115)	(0.117)		(0.081)	(0.078)
Wid/Div/Sep		1.15	1.17		1.22	1.25
		(0.247)	(0.253)		(0.235)	(0.242)
Education (ref: high school or less)		0.98	0.99		0.96	0.98
		(0.097)	(0.099)		(0.085)	(0.086)
Income (ref: less than 1M won)						
1-3M Won		0.73*	0.82		0.92	0.92
		(0.108)	(0.122)		(0.121)	(0.122)
3-5M Won		0.78+	0.85		0.90	0.91
		(0.119)	(0.130)		(0.122)	(0.123)
5-8M Won		0.94	1.05		0.81	0.82
		(0.152)	(0.171)		(0.116)	(0.118)
More Than 8M Won		0.85	0.99		0.95	0.96
		(0.163)	(0.194)		(0.162)	(0.166)
Religion (ref: none)						
Protestant		1.00	1.01		1.31**	1.31**
		(0.098)	(0.100)		(0.113)	(0.114)
Buddhist		0.77*	0.75*		1.19+	1.14
		(0.086)	(0.085)		(0.115)	(0.111)
Catholic		1.08	1.05		1.37**	1.33*
		(0.137)	(0.135)		(0.159)	(0.154)
Other		1.09	1.07		1.42+	1.41+
		(0.252)	(0.247)		(0.283)	(0.280)
Political Liberalism		1.01	1.02		0.96*	0.96*
		(0.021)	(0.022)		(0.018)	(0.018)
Worry of COVID-19 (ref: not worried)						
Somewhat Worried			1.13			1.36**
			(0.142)			(0.148)
Worried a Lot			1.68**			1.65**
			(0.252)			(0.217)
Perceived Likelihood of COVID-19 Infection (ref: not likely)						
Somewhat Likely			0.52**			1.12
			(0.049)			(0.093)
Very Likely			0.74+			1.41*
			(0.131)			(0.223)
COVID-19 Symptoms (ref: no)			0.73			1.18
			(0.157)			(0.226)
COVID-19 Test (ref: no)			0.86			0.89
			(0.137)			(0.126)
COVID-19 Positive (ref: no)			0.14**			0.62
			(0.067)			(0.257)
Observations	3,032	3,032	3,032	3,032	3,032	3,032

Exponentiated standard errors in parentheses

** p<0.01

* p<0.05

+ p<0.1

**Fig 3:**
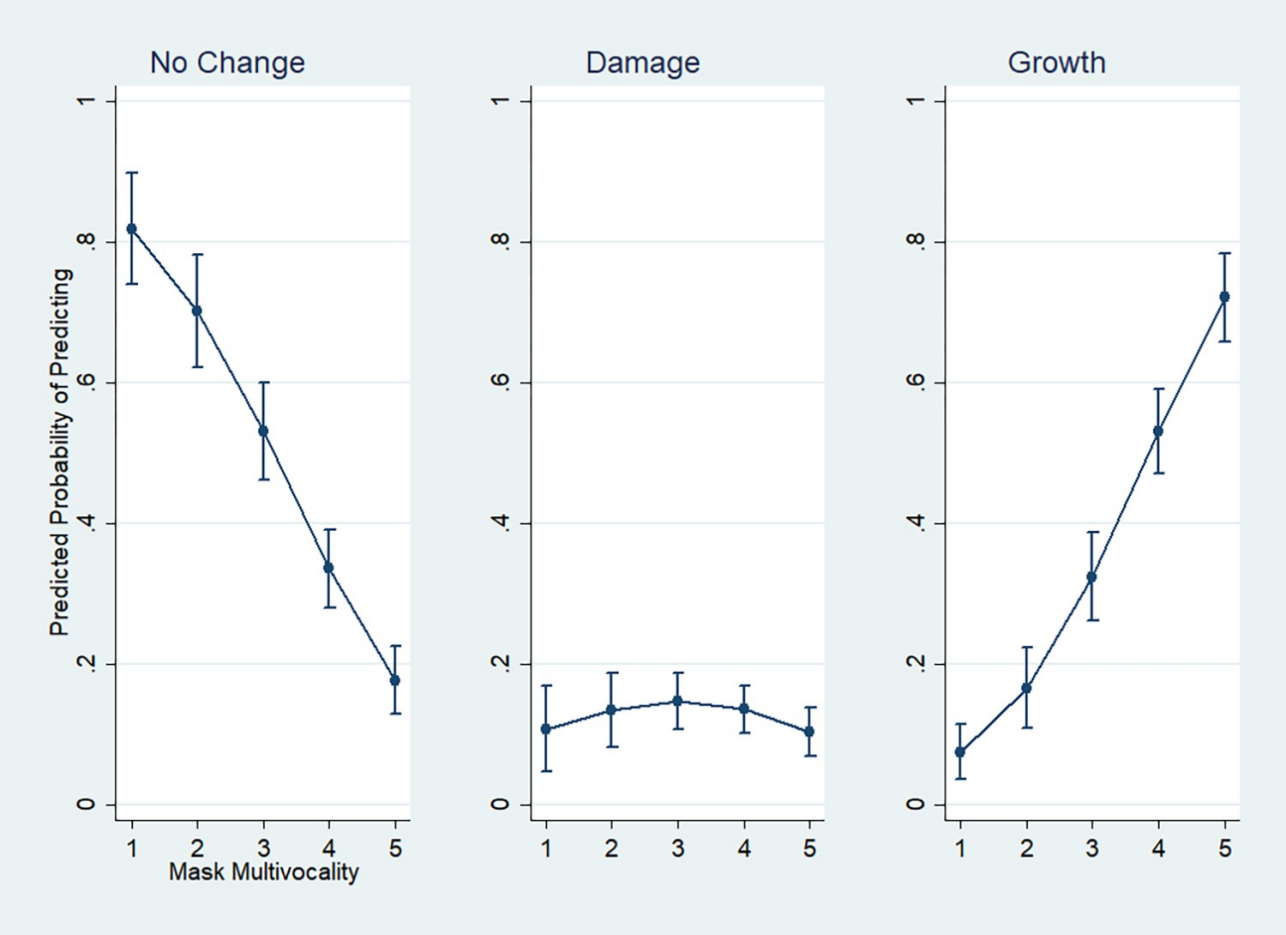
Predicted probabilities of anticipating different futures in the aftermath of the covid-19, depending on mask multivocality. Probabilities (dots with 95% CI ticks) are predicted for a person who holds modal values in gender (male), age (40s), residence (a metropolitan area), marital status (married), education (college or more), religion (non-religionist), household income (3-5M won), political liberalism (6 out of 10), COVID-19 worry (worried a little), COVID-19 likelihood (a little likely), COVID-19 symptoms (no), COVID-19 test (no), and COVID-19 positive (no).

Table 5 addresses Hypothesis 3–2 by using people’s outlook on the post-COVID-19 social changes. The dependent variable is a nominal variable measuring whether people anticipate no change, post-pandemic growth, or post-pandemic damage. The response category ‘damage’ is set as the reference. The models produce a robust pattern. In the full Model 3, when respondents ascribe more meanings to the mask by a unit, the probability for them to anticipate growth versus damage increases by 78% (= 1.78–1.00), and the probability to anticipate no change versus damage decreases by 31% (= 1.00–0.69). For ease of interpretation, Fig 3 reports probability changes in each of the three response categories, depending on mask multivocality. This prediction is based on Model 3 of Table 5. Three panels show that mask multivocality has no effect on respondents’ anticipation of damage; it has a positive effect on the anticipation of growth and a negative effect on the anticipation of no change. It suggests that respondents who place multiple meanings to the mask are less likely to anticipate no change in the aftermath of the pandemic; in other words, they are likely to expect changes and transformations in society. Regarding which kinds of changes, they anticipate new growth rather than damage.

**Table 5:** Relative rate ratios of predicting post-covid-19 social changes (no change or growth vs. damage) from multinomial logistic regression models.

	Model1		Model2		Model3	
	No Change	Growth	No Change	Growth	No Change	Growth
Multivocality of the Mask	0.60**	1.56**	0.63**	1.60**	0.69**	1.78**
	(0.060)	(0.157)	(0.065)	(0.169)	(0.071)	(0.193)
Gender (ref: male)			0.72**	0.67**	0.71**	0.66**
			(0.072)	(0.067)	(0.073)	(0.068)
Age (ref: 20s)						
30s			0.97	1.48*	0.99	1.49*
			(0.151)	(0.237)	(0.155)	(0.240)
40s			1.15	1.92**	1.09	1.77**
			(0.195)	(0.334)	(0.187)	(0.311)
50s			1.01	1.14	0.96	1.05
			(0.185)	(0.215)	(0.178)	(0.200)
60s or More			0.92	1.38	0.85	1.22
			(0.208)	(0.319)	(0.197)	(0.287)
Place of Residence (ref: metropolis)						
Suburb			0.91	1.00	0.88	0.97
			(0.115)	(0.127)	(0.112)	(0.125)
Small City			0.93	1.04	0.90	1.01
			(0.110)	(0.123)	(0.108)	(0.120)
Rural			1.02	1.09	0.97	1.01
			(0.238)	(0.258)	(0.229)	(0.243)
Marital Status (ref: single)						
Married			0.84	1.03	0.88	1.10
			(0.115)	(0.140)	(0.122)	(0.152)
Wid/Div/Sep			0.74	1.07	0.73	1.06
			(0.206)	(0.289)	(0.205)	(0.289)
Education (ref: high school or less)			1.16	1.37*	1.14	1.34*
			(0.141)	(0.170)	(0.140)	(0.170)
Income (ref: less than 1M won)						
1-3M Won			1.18	1.22	1.16	1.22
			(0.210)	(0.225)	(0.209)	(0.230)
3-5M Won			1.30	1.51*	1.27	1.50*
			(0.241)	(0.289)	(0.238)	(0.291)
5-8M Won			1.55*	1.55*	1.52*	1.55*
			(0.308)	(0.317)	(0.305)	(0.321)
More Than 8M Won			1.18	1.16	1.13	1.16
			(0.276)	(0.283)	(0.268)	(0.287)
Religion (ref: none)						
Protestant			0.81+	0.86	0.82	0.88
			(0.102)	(0.109)	(0.104)	(0.112)
Buddhist			0.85	0.86	0.90	0.93
			(0.120)	(0.122)	(0.129)	(0.134)
Catholic			0.63**	0.72*	0.65**	0.76+
			(0.101)	(0.114)	(0.107)	(0.122)
Other			1.84+	1.63	1.96*	1.76+
			(0.590)	(0.537)	(0.636)	(0.593)
Political Liberalism			1.09**	1.30**	1.09**	1.30**
			(0.030)	(0.036)	(0.030)	(0.036)
Worry of COVID-19 (ref: not worried)						
Somewhat Worried					0.78	0.66*
					(0.135)	(0.116)
Worried a Lot					0.46**	0.40**
					(0.092)	(0.079)
Perceived Likelihood of COVID-19 Infection (ref: not likely)						
Somewhat Likely					0.88	0.83
					(0.108)	(0.103)
Very Likely					0.66+	0.59*
					(0.143)	(0.130)
COVID-19 Symptoms (ref: no)					1.25	0.96
					(0.325)	(0.270)
COVID-19 Test (ref: no)					0.98	0.93
					(0.195)	(0.189)
COVID-19 Positive (ref: no)					1.17	0.53
					(0.688)	(0.386)
Constant	10.85**	0.27**	5.98**	0.03**	7.08**	0.04**
	(4.225)	(0.109)	(2.739)	(0.014)	(3.347)	(0.019)
Observations	3,032	3,032	3,032	3,032	3,032	3,032

Exponentiated standard errors in parentheses

** p<0.01

* p<0.05

+ p<0.1

Drawing on the previous pattern, Table 6 examines how mask multivocality relates to people’s attitudes toward two exemplary changes that they currently encounter: social distancing measures and the advocacy movement for natural environments amid the pandemic. As the two dependent variables record responses on a 5-point ordinal scale, Table 6 reports the result from the ordered logistic regression models. Additional robustness analyses with the OLS regression and generalized ordered logistic regression models (available from the author upon request) agree with the result in Table 6. People who take more meanings from the mask tend to anticipate that social distancing measures newly introduced during the pandemic will be in place after the pandemic. They also expect that people will consider conserving natural environments more than harnessing them for economic development.

**Table 6:** Odds ratios from the ordered logistic models of social distancing in place (models 1 to 3) and natural conservation in place (models 4 to 6) regressed upon the multivocality of the mask and other covariates.

	Model1	Model2	Model3	Model4	Model5	Model6
	DV: Social Distancing in Place			DV: Natural Conservation in Place		
Multivocality of the Mask	2.11**	2.07**	2.00**	1.41**	1.38**	1.34**
	(0.152)	(0.154)	(0.151)	(0.097)	(0.099)	(0.097)
Gender (ref: male)		1.05	1.06		1.14+	1.11
		(0.074)	(0.075)		(0.079)	(0.077)
Age (ref: 20s)						
30s		0.96	0.95		0.98	0.98
		(0.107)	(0.107)		(0.108)	(0.108)
40s		1.04	1.07		1.45**	1.50**
		(0.124)	(0.129)		(0.171)	(0.177)
50s		0.99	1.02		1.70**	1.74**
		(0.130)	(0.135)		(0.219)	(0.225)
60s or More		0.97	0.99		1.76**	1.79**
		(0.158)	(0.163)		(0.281)	(0.291)
Place of Residence (ref: metropolis)						
Suburb		0.90	0.92		0.88	0.89
		(0.081)	(0.082)		(0.077)	(0.078)
Small City		0.89	0.90		0.88+	0.88
		(0.073)	(0.075)		(0.071)	(0.071)
Rural		1.02	1.06		1.01	1.04
		(0.171)	(0.178)		(0.165)	(0.172)
Marital Status (ref: single)						
Married		1.14	1.12		1.14	1.13
		(0.107)	(0.106)		(0.106)	(0.105)
Wid/Div/Sep		1.01	1.00		0.94	0.94
		(0.193)	(0.192)		(0.177)	(0.176)
Education (ref: high school or less)		1.19+	1.20*		0.93	0.92
		(0.105)	(0.107)		(0.080)	(0.079)
Income (ref: less than 1M won)						
1-3M Won		0.86	0.87		0.83	0.85
		(0.115)	(0.117)		(0.109)	(0.113)
3-5M Won		0.95	0.98		0.80+	0.81
		(0.131)	(0.135)		(0.108)	(0.110)
5-8M Won		0.88	0.91		0.67**	0.68**
		(0.128)	(0.132)		(0.096)	(0.098)
More Than 8M Won		1.07	1.10		0.78	0.80
		(0.188)	(0.194)		(0.133)	(0.137)
Religion (ref: none)						
Protestant		1.16+	1.14		1.28**	1.29**
		(0.102)	(0.101)		(0.111)	(0.112)
Buddhist		1.00	0.96		1.18+	1.15
		(0.099)	(0.096)		(0.115)	(0.112)
Catholic		1.37**	1.34*		1.09	1.07
		(0.162)	(0.158)		(0.126)	(0.124)
Other		1.48+	1.45+		1.24	1.21
		(0.300)	(0.297)		(0.241)	(0.234)
Political Liberalism		1.02	1.03		1.07**	1.07**
		(0.019)	(0.020)		(0.020)	(0.020)
Worry of COVID-19 (ref: not worried)						
Somewhat Worried			1.01			1.31*
			(0.112)			(0.144)
Worried a Lot			1.27+			1.78**
			(0.171)			(0.237)
Perceived Likelihood of COVID-19 Infection (ref: not likely)						
Somewhat Likely			1.00			0.87+
			(0.084)			(0.071)
Very Likely			1.33+			0.72*
			(0.222)			(0.117)
COVID-19 Symptoms (ref: no)			1.02			1.15
			(0.202)			(0.218)
COVID-19 Test (ref: no)			1.19			1.06
			(0.175)			(0.147)
COVID-19 Positive (ref: no)			0.75			1.10
			(0.329)			(0.461)
Observations	3,032	3,032	3,032	3,032	3,032	3,032

Exponentiated standard errors in parentheses

** p<0.01

* p<0.05

+ p<0.1

## Conclusion and discussion

This study has initiated the current inquiry with an empirical surprise about Koreans’ intriguing practices with the face mask against the COVID-19 pandemic. The surprise is not that Koreans, along with other East Asians, are willing to wear the mask unlike Westerners, but that there are contradictions and inconsistencies in their mask practices. Contradictions and inconsistencies would not necessarily invite a serious sociological inquiry, however, unless they exist among people’s devotion, commitment, and concerns with the matter at hands. Therefore, the surprise in due terms regards the presence of contradictions and inconsistencies among very agentic individuals about a life matter.

Against this background, it is necessary to make a theoretical detour to address what committed and agentic individuals (i.e., agency and individuality) mean in the sociological literature and how agency and individuality are ever related to inconsistencies and contradictions. For this purpose, this study has collected a group of studies under the contemporary title of the sociology of “social action” [e.g., 19] or the “action theory” of agency [e.g., 17, 18], in which the matter of human agency is theorized in terms of action and its innate multiplicity. Action theories state that action, or human agency proper, is composed of multiple elements: individual and supra-individual, rational and emotional, and human and nonhuman. Thus comes the notion of individuals proper as social individuals who found their individuality upon supra-individual as well as individual elements.

Only within this appreciation of multiplicity can the empirical observation of inconsistencies be incorporated and given due attention. Action theories then give the initial empirical surprise an immediate, formal theoretical reformulation. Human agency, whether in Korea or not, is innately multi-faceted and potentially contradictory and inconsistent within it. This conceptual reformulation suffices to be a quick answer to the surprise. Koreans are self-contradictory and inconsistent in mask practices because they are meaningfully agentic individuals. The only remaining matter is how to qualify these agentic individuals in real terms. Drawing on the literature on social individuals, therefore, this study has shown that these agentic individuals are grouped into social individuals of different shapes in Korea, such as atomists, collectivists, and dualists. This elaboration of agentic individuals (i.e., agency and individuality) into three types of social individuals marks the first contribution of this study.

Yet, current action theories aim more, and mask practices have more details in the empirical context. In asserting multiplicity and possible contradictions, action theories are additionally concerned with theorizing in more specific terms how those multiple elements of agency are organized in action and what this organizing process reveals about human agency and humanity. This study has set the empirical investigation of mask practices as a test bed to address these concerns. Therefore, it draws on another group of studies with specific theoretical languages for mask practices. This expansion of action theory to interests in the mask makes another contribution. This study suggests that action, as human agency proper, can be meaningfully calibrated in the mask, such as surgical face masks in the current pandemic.

This study soon realizes that this sort of setup, or the transmission of the interest in individuality into the interest in the mask (if not surgical face masks yet), is not unprecedented. Only more novel contributions have yet to be made. Among these precedents are the sociology of the mask [36, 37] and the anthropology of the mask [30, 38]. These studies and subsequent inquiries address the mask as an ongoing practice (rather than a thing or a reified depiction) of the self, identity, subjectivity, individuality, and agency. They highlight that the mask is a constituent part of individuality on a par with other constituents like an individual body and supra-individual spirits. They further suggest that the mask itself is individuality and these other constituents should be reformulated as different kinds of the mask: for example, the Goffmanesque equal treatment of the mask and the face and the equality of the frontstage and the backstage; the Maussian total treatment of the mask simply as the person (i.e., the mask as an instance of the total humanity). Lastly, the mask as individuality delivers multiple elements, such as its wearer in its body/soul, its surrounding others and natural/super-natural entities, the past, and the present. Once the mask is a composite of multiple elements, it becomes a dynamic process, or simply a masquerade, the collective social drama. Ultimately, this view predicts that the face mask against COVID-19 signifies multiple elements at once. This study has qualified this prediction by demonstrating that Koreans take the face mask as signifying alters and ego at once; physical protection, psychological relief, symbolic social ritual at once; and both life adversity and life betterment during the pandemic. This elaboration is another contribution of this paper. It argues that the inconsistencies of Koreans’ mask practices are embedded in and reveal tensions and overlaps among these multiple meanings.

However, this view of the mask sweepingly as a masquerade and a process of multiple elements is a conceptual exaggeration, although it duly adds to the sociological imagination of individuality (i.e., individuality properly as a process of multiple elements) to which reality often corresponds. The fact that the mask refers to multiple elements does not necessarily lead to the realization of the mask as a dynamic process. Analytically speaking, multiple elements do not necessarily beget the dynamism of one element being (re-)assembled with another unrestrictedly nor the vitality of all available elements interacting with one another without any outliers. In addition, they do not promise the dynamism of unknown, novel elements emerging from old ones. To the extent to which one element is arranged with another in a rather fixed manner, to the extent to which only some and not all elements interplay with one another, or to the extent to which only existing and no emergent elements come into play, the dynamism that we expect from the social world as a process and a flow [35 for the social life-process, 125: 385 for a process underlying forms, 126 for totality as a persistent process, 127] becomes qualified. To these extents, the multiplicity of the mask does not realize itself to its full multivocality, resulting instead in the different degrees and shapes of multivocal processes. Therefore, a due sociological task is to examine how the mask, as one specific kind in the social world as a process, ends up being certain types and patterns. This is what the existing sociological and anthropological works of the mask leave for subsequent inquiries like this paper.

This view that the mask should be understood as a full course of masquerade, including multiple elements, holds an eminent place in the literature, which yet awaits qualifications and elaborations in a variety of real-world experiences. In the literature, the mask affords a system of many social and natural (sub-)systems to its wearers, inclusive of a wearer’s intra-individual system (bodily or mental) [30, 38], and a masquerade becomes a multi-faceted event for the participant to become a meaningful self.

This paper further argues that participation in such a masquerade is full of tensions, suspicions, and even doubts. It is a “test” in which an individual mask-wearer needs to show “the presence within him an element of an impersonal force, or of the ancestor, or of the personal god, in any case of the superhuman power, spiritual and ultimate” (or supra-individual authorities like a nation-state) and, only then, becomes absorbed in the system as a legitimate individual [9, 30: 6]. Mauss himself, however, does not provide further qualifications of the masquerade where, we know, but Mauss did not highlight, some pass the test while others fail it. Although it is these present-day wearers who give material and sensible forms to (and thus enliven) the otherwise immaterial spirits and powers from the past [112], the literature is indifferent to further qualifications of how this enlivenment succeeds or fails.

The literature [38] acknowledges that the mask produces contradictions between its wearer and the outside, between the wearer’s face and the mask façade/materiality, and between an individual wearer and a social system; then, Levi-Strauss immediately concludes that the mask resolves these contradictions as well. There is no temporal or political lapse in Levi-Strauss between when the mask produces contradictions and when it resolves these contradictions. All contradictions are resolved readily, which is reminiscent of Parsons’ view of human agency as “efforts” to solve problems among different systems (i.e., “control” problems) and yet his little treatment of the efforts thereafter [25]. What needs to follow is to further qualify the extent to which and the ways in which the contradictions endemic to the mask are resolved by the same mask.

One way to qualify varying degrees of the processual realization and concretization [17: 163] of the mask as a masquerade is to discern the variable multivocality of the mask in one way or another. The existing works on the mask, while deficient, are not without clues for this task. Among many insights from the literature, the most important is that the mask is worn and practiced at the very intersections between individual and supra-individual elements, whether they are material or symbolic. This insight suggests that the task can reasonably draw on the types of social individuals (i.e., atomists, collectivists, and dualists) that this paper has shown emerge at the corresponding intersections of individual and supra-individual elements in the general action theory.

Therefore, this paper has attempted to associate the multiple meanings of the mask among Koreans (i.e., mask multivocality) with the three types of social individuals. This schematic yet theoretically driven exercise has succeeded in revealing patterns between the semiotic concretization of the mask and the typology of social individuality. Based on this exercise, it argues that the dynamic and inconsistent practices of the mask among Koreans are patterned into those of atomist, collectivist, and dualist social individuals. What the current COVID-19 masquerade reveals includes three different ways in which social individuals exist in Korea. What underlies the self-contradictory practices of the mask are various ways in which individuals make and remake their individuality, which is an awaited contribution to the sociology of the mask.

Against this backdrop of the patterned association between the mask and social individuals, this study argues for one self-revealing nature of humanity in action: multivocality that the sociology of action has long attended to. On the basis of the empirical evidence of the mask, this study agrees with existing action theories on the conclusion that agency and individuality are in multivocality. Subsequently, it has attempted to address an additional question on what multivocality means in more specific terms by investigating concrete experiences of the face mask in the current pandemic. By showing the statistical association that Koreans ascribing more voices to the mask are more susceptible to new promises as well as damages from the pandemic, it argues, first, that multivocality is manifested as both vulnerability and transformative power and, second, that these two aspects are closely knit together. While the former statement elaborates on what the multivocality of humanity means, the latter further suggests that it is the vulnerability in humanity that enables the transformative power in human agency; vulnerability is implied in the transformative and creative power of humanity. These statements mark the final contribution to action theory in general, dipped from the Korean experiences of the COVID-19 masquerade. Humans are as transformative as they are vulnerable.

As this study carries these contributions to a global level, there can be a controversy about whether inconsistencies and contradictions in mask practices are generalizable beyond the Korean or Asian context. It is not uncommon to read studies about people outside Asian countries who customarily align the face mask during the pandemic only with the Chinese, Korean, Japanese, or (East-)Asian ethnicity [2, 98, 128, 129]. This study hesitates to delimit these experiences only to one country or a region, based on still-evolving reports of confusions and contradictions from different areas of the world [11, 14, 15, 130]. Studies report not only the prevalence of the mask among non-Asians but also contradictions and inconsistencies in their mask practices. For the US, a national survey shows that a majority (80–90%) of Americans are wearing the mask in public spaces when it is not mandated by local governments [16], while Americans are reported to vehemently verbalize their disproof of the idea of mask-wearing [131–135]. These accounts are only the flip side (of the same coin) of Koreans who generally accept the mask mandate and often fail to (and sometimes intend not to) keep it in practice. An observational study of about 10,000 shoppers in retail stores in the US reports that the percentage of mask-wearers soared from 41% in June to over 90% in July immediately after the governmental mandates for mask-wearing [136], which is analogous to Koreans’ sudden and ‘opportunistic’ [118] acceptance of mask-wearing and overnight hassles resulting in supply shortages across the country. Despite possible variations in the extent of inconsistent and contradictory practices between Asian and non-Asian populations, and even between different Asian countries, it seems to be generalizable outside Korea that people are engaged in multiple and often contradictory practices of the face mask during the current pandemic.

The finding in this study that there are different types of social individuals among Koreans under the face mask (i.e., atomists and dualists as well as collectivists) renews the debate among existing studies of social individuality among Koreans or East Asians. In particular, studies have asserted that the relatively ready acceptance of the face mask against the current as well as the past episodes of viral infections (e.g., SARS and influenzas) in East Asian countries is attributable to their collectivist culture that puts a greater emphasis on the community at large than individuals who are to be effaced by the mask [2, 101, 137]. A counter-argument stresses not the collectivist culture but newly adopted, modern individualist cultures that use the mask as an efficient isolating, decentering device [100, 138]. In their sweeping, binary arguments, the former overlooks people in the region who are concerned only with themselves and not collectivities; the latter ignores the unmistakable existence of collectivists. More importantly, the former is negligent of people who pursue collectivities often for the sake of individual welfare, while the latter is silent about people who seek individual existence by way of collectivities. The current typology of social individuals in this study provides a balanced conceptual alternative that does not pre-determine East Asians to be either binary only (i.e., collectivists or atomists) but allows them to be collectivists, atomists, or both at once. In addition, its theorization of the mask as having multiple voices loosens the rigid conceptual codification of mask practices as either collectivist only or atomist only. Instead, the mask can be atomist as well as collectivist at once; dividing as well as bridging; material as well as symbolic; transformative as well as vulnerable. Given this two-pronged conceptual reformulation and subsequent empirical affirmation, while it seems to be true that East Asians are more ready to accept the mask than others, it is not their being simply collectivists or atomists that accounts for the acceptance. Instead, they wear the mask as collectivists, atomists, or dualists. In Korea, there are as many dualists as collectivists. They wear the mask, being transformative as well as vulnerable social individuals. By the same token, we cannot simply assume atomist individualism or aggressive self-assertion among Americans and other non-Asians who oppose the mask. Their opposition may well reflect collectivist individualism and vulnerable self-subsistence. To these effects, cross-national inquiries on the local makeup of social individuals and the semiotic concretization of the mask have yet to be made.

Lastly, speaking of the semiotics of the mask or the sociology of the mask, can it be a general and genuine sociological venture? There is a significant tradition in place. The sheer material and symbolic affordance of the mask has been aptly described by a renowned anthropologist’s question on why the masks of American Indians are in the “unusual shapes” and “so ill-adapted to their function” [38: 12]. The mask is “diacritical” and an essential part of various social systems, he answers [38: 93]. This intimate fact that the mask is afforded to us, and we are afforded to the mask in the world is recognized earlier by Mauss. He states that we are concerned about the mask because it is believed to be part of the “very general, and probably the very primitive, form of the notion of cause,” such as mana, magic, time, space, the whole, genus, and the mind [30: 1–2]. In addition, the mask is as essential as the face to acting and practice in everyday life [36, 37]. In this tradition, the mask has been with us for long, not only in times of emergency like the current pandemic but in ceremonial rituals and everyday routines, providing opportunities in which the self and individuality intimately intersect with systems and sociality. At the intersection, non-structure emerges together with structure; absence comes in the shape of presence, and vice versa; emotion and corporeality come with cognition and mentality. Students of action theories who aim to articulate the social–individual (not simply the social or the individual) and its multiplicity, or how the social world as a process makes patterned states (and how patterns constitute the process), have only to further investigate instances of the mask in different contexts. There is a decent record of contemporary empirical studies in cultural studies of indigenous people, contemporary arts, public health, gender studies, and power/politics. This paper hopes future inquiries to bear more with the general action theoretical undercurrents presented here. Instances of the intriguing coalescence of the COVID-19 masks for health with the political protests for freedom, such as the “I am not a virus” masks worn for Asians [139, 140], the “I can’t breathe” masks worn for the Black Lives Matter movement [141–143], and the same “I can’t breathe” chants in anti-mask political campaigns [144, 145], imply that a comprehensive perspective from action theory, that is ready to resonate with contradictions and collaborations in the mask at once, is necessary for properly appreciating the mask that stays with us in varying forms. Everyday life is a social drama, a face-work, a mask-work, or a masquerade around ultimate forces and powers inside and outside COVID-19.
